# Optimization of a modeling platform to predict oncogenes from genome‐scale metabolic networks of non‐small‐cell lung cancers

**DOI:** 10.1002/2211-5463.13231

**Published:** 2021-07-20

**Authors:** You‐Tyun Wang, Min‐Ru Lin, Wei‐Chen Chen, Wu‐Hsiung Wu, Feng‐Sheng Wang

**Affiliations:** ^1^ Department of Chemical Engineering National Chung Cheng University Chiayi Taiwan

**Keywords:** cancer cell metabolism, constraint‐based modeling, flux balance analysis, tissue‐specific metabolic models, trilevel optimization

## Abstract

Cancer cell dysregulations result in the abnormal regulation of cellular metabolic pathways. By simulating this metabolic reprogramming using constraint‐based modeling approaches, oncogenes can be predicted, and this knowledge can be used in prognosis and treatment. We introduced a trilevel optimization problem describing metabolic reprogramming for inferring oncogenes. First, this study used RNA‐Seq expression data of lung adenocarcinoma (LUAD) and lung squamous cell carcinoma (LUSC) samples and their healthy counterparts to reconstruct tissue‐specific genome‐scale metabolic models and subsequently build the flux distribution pattern that provided a measure for the oncogene inference optimization problem for determining tumorigenesis. The platform detected 45 genes for LUAD and 84 genes for LUSC that lead to tumorigenesis. A high level of differentially expressed genes was not an essential factor for determining tumorigenesis. The platform indicated that pyruvate kinase (PKM), a well‐known oncogene with a low level of differential gene expression in LUAD and LUSC, had the highest fitness among the predicted oncogenes based on computation. By contrast, pyruvate kinase L/R (PKLR), an isozyme of PKM, had a high level of differential gene expression in both cancers. Phosphatidylserine synthase 1 (*PTDSS1*), an oncogene in LUAD, was inferred to have a low level of differential gene expression, and overexpression could significantly reduce survival probability. According to the factor analysis, *PTDSS1* characteristics were close to those of the template, but they were unobvious in LUSC. Angiotensin‐converting enzyme 2 (*ACE2*) has recently garnered widespread interest as the SARS‐CoV‐2 virus receptor. Moreover, we determined that *ACE2* is an oncogene of LUSC but not of LUAD. The platform developed in this study can identify oncogenes with low levels of differential expression and be used to identify potential therapeutic targets for cancer treatment.

## Graphical abstract

Identifying differentially expressed genes is critical in exploring molecular mechanisms of cancers. A high level of differential gene expression is not an essential factor for tumorigenesis. We developed an optimization platform that can identify oncogenes with low levels of differential expression and potential therapeutic targets for cancer treatment. The platform was applied on LUAD and LUSC to illustrate its performance.

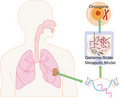

**Abbreviations**
ACAA2: acetyl‐CoA acyltransferase 2
ACE2: angiotensin‐converting enzyme 2
BCAT1: branched chain amino acid transaminase 1
COBRA: constraint‐based reconstruction and analysis toolbox
CORDA: cost optimization reaction dependency assessment
COSMIC: Catalogue Of Somatic Mutations In Cancer
DEG: differentially expressed gene
ENO1: enolase 1
FVA: flux variability analysis
GLO1: glyoxalase I
GPR: gene–protein–reaction
GSMN: genome‐scale metabolic network
HPA: human protein atlas
iMAT: integrative metabolic analysis tool
LUAD: lung adenocarcinoma
LUSC: lung squamous cell carcinoma
MFVA: metabolite‐flow variability analysis
NCI‐60: 60 human tumor cell line anticancer drug screen from the US National Cancer Institute
NF‐κB: nuclear factor‐κB
NHDE: nested hybrid differential evolution
NSCLC: non‐small‐cell lung carcinoma
PKLR: pyruvate kinase L/R
PKM: pyruvate kinase
PSPH: phosphoserine phosphatase
PTDSS1: phosphatidylserine synthase 1
SARS‐CoV‐2: severe acute respiratory syndrome coronavirus 2
SHMT1: serine hydroxymethyltransferase 1
SLC: solute carrier
TCGA: The Cancer Genome Atlas
TLOP: trilevel optimization problem
VMH: Virtual Metabolic Human

Lung carcinoma is one of the most common malignancies, resulting in the largest number of cancer‐related deaths worldwide [1]. Two main subtypes of lung cancer exist, namely small‐cell lung carcinoma and non‐small‐cell lung carcinoma (NSCLC), accounting for 15% and 85% of all lung cancers, respectively [2]. Lung adenocarcinoma (LUAD) and lung squamous cell carcinoma (LUSC), two main subtypes of NSCLC, are predominant lung cancers, accounting for 40% and 33%, respectively, of cancer deaths worldwide [3]. Lung carcinoma is related to genetic and epigenetic dysregulation, and understanding its biological mechanism is crucial for developing effective treatment.

Systems biology approaches and big database mining have been applied to construct genetic and epigenetic networks with next‐generation sequencing data of LUAD and LUSC for comparing the differential genetic and epigenetic progression mechanisms [4]. Such approaches might enable the deciphering of genotype discrepancy for both tissues. However, genes and their expression alone do not always constitute a reliable indicator of cellular phenotype. The recent availability of omics datasets allows the analysis of cellular characteristics at the levels of genes, mRNAs, proteins, and metabolites. A genome‐scale metabolic network (GSMN) can offer a biological mechanism that links genotype to phenotype; it can help us understand cell physiology and certain disease phenotypes caused by metabolic dysregulation [5, 6]. Human metabolism is complex and specialized in different tissue and cell types. The reprogramming of tissue‐specific metabolism in GSMNs will provide deeper insights into the metabolic basis of various physiological and pathological processes. Such metabolic reprogramming approaches have been applied to predict oncogenes, essential enzymes, and drug targets for developing novel medical treatments [7, 8, 9, 10, 11].

Cancer metabolism is an emerging hallmark of cancer [12]. Genetic alterations and epigenetic modifications of cancer cells result in the abnormal regulation of cellular metabolic pathways that differ from normal cells. GSMNs combined with constraint‐based modeling approaches can predict the metabolic reprogramming of cancer cells to reveal oncogenes, essential enzymes, and drug targets for developing novel medical treatments [13, 14, 15, 16, 17, 18, 19, 20, 21, 22]. The first large‐scale reconstructed metabolic model for cancer was built based on the gene expression data of all cancer cell lines in the NCI‐60 collection and the human general metabolic network (Recon 1) [14, 23]. This model was applied on the identification of essential genes and cytostatic drug targets of cancer cell lines [14] and prediction of metabolic targets for inhibiting cancer migration [23]. The GSMN (*iHepatocytes2322*) for hepatocytes was reconstructed by extending Recon 1 using data from Human Metabolic Reaction 2.0 database and proteomics data in Human Protein Atlas (https://metabolicatlas.org/). This GSMN was used to identify PSPH, SHMT1, and BCAT1 as potential therapeutic targets for the treatment of nonalcoholic steatohepatitis using the transcriptomics data obtained from patients with nonalcoholic fatty liver disease [16]. Another small‐scale constraint‐based model was created and combined with machine‐learning techniques to investigate the mechanism of pyruvate dehydrogenase under hypoxia [13].

Due to the complexity and specialization of human metabolism in different tissue and cancer cells, mapping tissue‐specific metabolisms in GSMNs can advance the understanding of cancer metabolism [24]. Recon 2.2 and Recon 3D are the most comprehensive human genome‐scale network reconstructions [25, 26]. Recon 2.2 was incorporated with the Human Protein Atlas (HPA) [27] to reconstruct GSMNs of the colorectal tissue [18] and head‐and‐neck tissue [21] at normal and cancerous states. Recon 2M.2 [28, 29] integrated with RNA‐Seq data from NCI‐60 cell lines presented a systematic framework for the generation of gene–transcript–protein–reaction that enables the accurate prediction of metabolic behaviors. Recon 3D is a human general genome‐scale network reconstruction that includes three‐dimensional metabolite and protein structure data and enables an integrated analysis of metabolic functions in humans. In this study, we first applied the CORDA method [30] integrated with The Cancer Genome Atlas (TCGA) database [31] and Recon 3D to reconstruct GSMNs for LUAD, LUSC, and their healthy cells. Multivariate analysis was used to analyze the reactions, metabolites, and enzyme‐encoding genes of these GSMNs to discriminate differential expressions between normal and cancer cells. The oncogene inference optimization formulation [18, 21] was used to mimic gene screening procedures in a wet laboratory to evaluate the mechanism by which gene dysregulations induce tumorigenesis.

## Materials and methods

### Reconstruction of tissue‐specific metabolic models

**Fig. 1:**
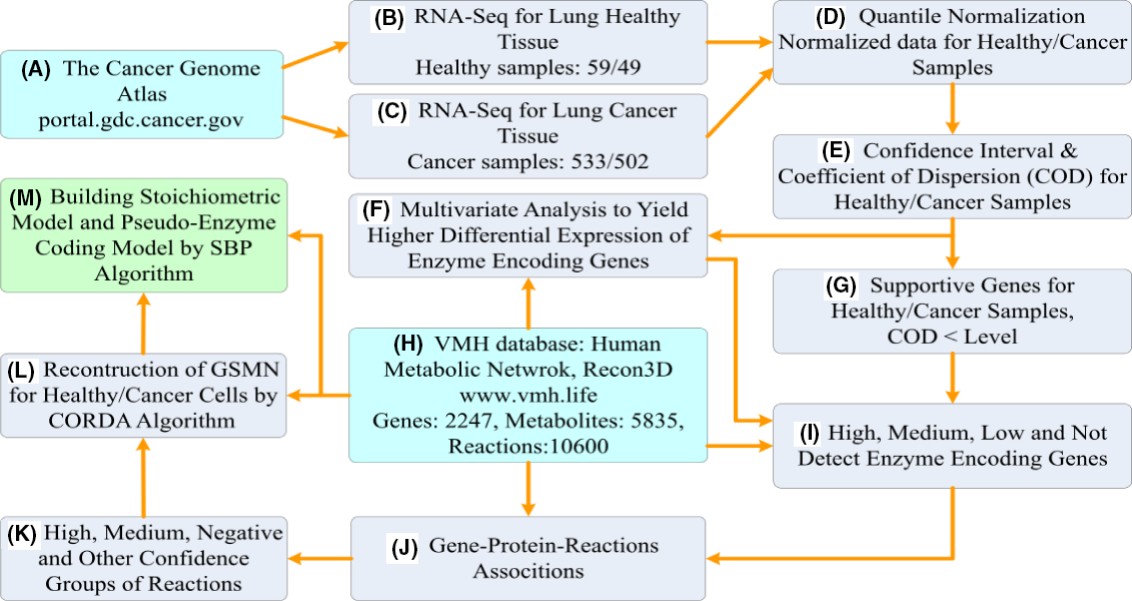
Roadmap of the reconstruction of genome‐scale metabolic models. Roadmap of the reconstruction of genome‐scale metabolic models for LUAD and LUSC and their corresponding healthy tissues. (A) Download RNA‐Seq data of LUAD and LUSC from the TCGA database. (B–G) Statistical analysis of download RNA‐Seq data to generate input information for the CORDA algorithm. (H) The general human GSMN (Recon 3D) was downloaded from VHM database (https://www.vmh.life) and used as a base model. (I) Classify enzyme‐encoding genes into four classes. (J) Compute gene–protein–reaction associations using the enzyme‐encoding genes and Recon 3D general model. (K) Identify reactions having various confidence indices. (L) Reconstruct a tissue‐specific metabolic model using the CORDA algorithm and Recon 3D general model. (M) Build the stoichiometric and GPR models in GAMS format for simulation.

This study applied RNA‐Seq data from TCGA database to reconstruct genome‐scale metabolic models (Fig. 1) for LUAD and LUSC and their corresponding healthy tissues. These metabolic models were entirely flux‐consistent inspected by the 'findFluxConsistentSubset' function of COBRA toolbox. In total, 533 and 502 samples for LUAD and LUSC and 59 and 49 corresponding healthy samples were collected, respectively. Quantile normalization was applied to normalize the raw data for healthy and cancerous samples to compute the mean, confidence interval, and coefficient of dispersion for each gene. Such data were then used to evaluate supportive genes and obtain a high differential expression of enzyme‐encoding genes between the cancer and healthy cells. Recon 3D consisted of 2247 enzyme‐encoding genes, which were classified into four levels based on their participation, namely high, medium, low, and not detected. Four groups of confidence reactions, namely high, medium, negative, and others, were obtained through gene–protein–reaction association in Recon 3D. The tissue‐specific GSMNs of healthy and cancer cells were reconstructed using the CORDA algorithm and saved in SBML format. We developed a systems biology program (SBP) platform to automatically develop the stoichiometric models and GPR model in GAMS format to perform the simulation.

### Oncogene inference optimization

The oncogene inference optimization framework was modified from a trilevel optimization problem (TLOP) that has been applied to analyze colorectal cancer [18]. In this study, the objectives in the TLOP considered the consistency between the change trends of mutant fluxes/metabolite flows compared with the template and that of cancer cells, and applied fuzzy equal constraints to quantitatively limit the change ratios of the mutant as close as possible to the template. These objectives in the modified approach can qualitatively and quantitatively optimize the flux pattern of a mutant as close as possible to the template. This oncogene inference optimization framework has been used to detect tumor suppressor genes in head‐and‐neck squamous cell carcinoma [21] and can be used as a mimic experiment for gene screening to predict oncogenes, similar to metabolic transformation algorithm for identification of drug targets [10, 32]. The outer optimization aims to infer the dysregulation of enzyme‐encoding genes that alter the metabolism of normal cells leading to cancer, and the inner optimization problems present perturbed behaviors of mutant cells. The mathematical formulation is expressed as follows:

(1) $$\left\{\begin{array}{l} \text{Outer optimization problem:}\\ \text{Similarity ratio of metabolite-flow rates and fluxes to the template:}\\ \max_{\delta ,z_{i}}SR_{M},\;\max_{\delta ,z_{i}}SR_{F}\\ \text{Fuzzy equal grade of metabolite-flow rates and fluxes}\\ \text{compared to the template:}\\ \tilde{\underset{\delta ,z_{i}}{\mathrm{Equal}}}\;LFC_{M}^{\mathrm{MUBL}}\approx LFC_{M}^{\mathrm{CABL}},\;\tilde{\underset{\delta ,z_{i}}{\mathrm{Equal}}}\;LFC_{F}^{\mathrm{MUBL}}\approx LFC_{F}^{\mathrm{CABL}}\\ \text{subject to the inner optimization problems:}\\ \left\{\begin{array}{l} \text{Flux balance analysis (FBA) problem}\\ \underset{v_{f/b}}{\max}obj\equiv \left(w_{\mathrm{ATP}}v_{\mathrm{ATP}}+w_{\mathrm{biomass}}v_{\mathrm{biomass}}\right)\\ \text{subject to}\\ N\left(v_{f}‐v_{b}\right)=0\\ v_{f/b,i}^{\mathrm{LB}}\leq v_{f/b,i}\leq v_{f/b,i}^{\mathrm{UB}},\;z_{i}\notin \Omega^{\mathrm{MU}}\\ v_{f/b,j}^{LB,MU}\leq v_{f/b,j}\leq v_{f/b,j}^{UB,MU},\;z_{j}\in \Omega^{\mathrm{MU}} \end{array}\right.\\ \left\{\begin{array}{l} \text{Uniform flux distribution (UFD) problem}\\ \underset{v_{f/b}}{\min}\sum_{i\in \Omega^{\mathrm{Int}}}{\left(v_{f,k}\right)}^{2}+{\left(v_{b,k}\right)}^{2}\\ \text{subject to}\\ N\left(v_{f}‐v_{b}\right)=0\\ v_{f/b,i}^{\mathrm{LB}}\leq v_{f/b,i}\leq v_{f/b,i}^{\mathrm{UB}},\;z_{i}\notin \Omega^{\mathrm{MU}}\\ v_{f/b,j}^{LB,MU}\leq v_{f/b,j}\leq v_{f/b,j}^{UB,MU},\;z_{j}\in \Omega^{\mathrm{MU}}\\ obj\geq obj^{*} \end{array}\right. \end{array}\right.$$

where *v*_f/b_ is the forward/backward flux vector of reversible reactions; **N** is an *m* × *n* stoichiometric matrix where *m* is the number of metabolites, and *n* is the number of reactions; $v_{f/b,i}^{\mathrm{LB}}$ and $v_{f/b,i}^{\mathrm{UB}}$ are the positive lower and upper bounds of the *i*th forward/backward flux, respectively; $v_{f/b,i}^{LB,MU}$ and $v_{f/b,i}^{UB,MU}$ are the positive lower and upper bounds of the *i*th upregulation, downregulation, or knockout flux in the set of mutated reactions **Ω** *^MU^* due to the *i*th enzyme dysregulation, which is determined using the GPR model; *obj* ^*^ is the maximum cellular objective obtained from the flux balance analysis (FBA) problem; $\tilde{\mathrm{Equal}}$ is the fuzzy equal objective function that represent the fuzzy goals. For example, the *LFC_m_^MUBL^* and *LFC_m_^CABL^* should be restored to a state that is as close as possible; the integer vector z is used to determine mutated enzymes; and δ is the regulated strength parameter for the mutants with a value within (0, 1].

The GPR model used a pseudo‐enzyme coding number strategy to represent GPR associations in Recon 3D [18]. It identified redundant pseudoenzymes and isozymes in the model such that the reactions were catalyzed through reduced association. Therefore, pseudoenzymes were applied to determine modulated genes, and the level of the mutated bounds was computed using the following equations:

(2) $$\begin{array}{c} \text{Upregulation:}\\ \left\{\begin{array}{l} \left(1‐\delta \right)v_{f,i}^{\mathrm{basal}}+\delta v_{f,i}^{\mathrm{UB}}\leq v_{f,i}\leq \;v_{f,i}^{\mathrm{UB}}\\ v_{b,i}^{\mathrm{LB}}\leq v_{b,i}\leq \left(1‐\delta \right)v_{b,i}^{\mathrm{basal}}+\delta v_{b,i}^{\mathrm{LB}};\;i\in \Omega^{\mathrm{MU}} \end{array}\right.\\ \text{Downregulation}:\\ \left\{\begin{array}{l} v_{f,i}^{\mathrm{LB}}\leq v_{f,i}\leq \left(1‐\delta \right)v_{f,i}^{\mathrm{basal}}+\delta v_{f,i}^{\mathrm{LB}}\\ \left(1‐\delta \right)v_{b,i}^{\mathrm{basal}}+\delta v_{b,i}^{\mathrm{UB}}\leq v_{b,i}\leq v_{b,i}^{\mathrm{UB}};\;i\in \Omega^{\mathrm{MU}}\backslash \Omega^{\mathrm{IZ}} \end{array}\right.\\ \left\{\begin{array}{l} v_{f,i}^{\mathrm{LB}}\leq v_{f,i}\leq v_{f,i}^{\mathrm{UB}}\\ v_{b,i}^{\mathrm{LB}}\leq v_{b,i}\leq v_{b,i}^{\mathrm{UB}};\;i\in \Omega^{\mathrm{MU}}\cap \Omega^{\mathrm{IZ}} \end{array}\right.\\ \text{Knockout}:\\ \left\{\begin{array}{l} v_{f,i}=0\\ v_{b,i}=0;\;i\in \Omega^{\mathrm{MU}}\backslash \Omega^{\mathrm{IZ}} \end{array}\right.\\ \left\{\begin{array}{l} v_{f,i}^{\mathrm{LB}}\leq v_{f,i}\leq v_{f,i}^{\mathrm{UB}}\\ v_{b,i}^{\mathrm{LB}}\leq v_{b,i}\leq v_{b,i}^{\mathrm{UB}};\;i\in \Omega^{\mathrm{MU}}\cap \Omega^{\mathrm{IZ}} \end{array}\right. \end{array}$$

where $v_{f/b.i}^{\mathrm{basal}}$ is the basal flux in the normal state, and **Ω** *^IZ^* is the set of reactions regulated by isozymes represented in the GPR model.

Multiple objectives are considered in the outer optimization problem in Eqn (1). In the first and second objectives, the similarity ratios of metabolite‐flow rates and fluxes (*SR_M_* and *SR_F_*) are maximized for determining a dysregulated metabolite‐flow/flux pattern that is as similar as possible to the template. The third and fourth objectives are used to obtain a mutant log2 fold change, $LFC_{M/F}^{\mathrm{MUBL}}$, of metabolite‐flow rates/fluxes as close as possible to that of the template, $LFC_{M/F}^{\mathrm{CABL}}$. The similarity ratios of the metabolite‐flow rates/fluxes (*SR_M_* and *SR_F_*) for a mutant are evaluated as follows:

(3) $$SR_{M/F}=\frac{\sum_{m=1}^{N_{M/F}}\left|\mu_{m}^{M/F}\right|}{N_{M/F}}$$

where the similarity indicator ($\mu_{m}^{M/F}$) for each metabolite‐flow rate or flux in the metabolic network is defined as follows:

(4) $$\mu_{m}^{M/F}=\left\{\begin{array}{l} 1,\;\text{if}\;LFC_{M/F,m}^{\mathrm{MUBL}}>tol_{+}\;\text{and}\;LFC_{M/F,m}^{\mathrm{CABL}}>tol_{+}\\ ‐1,\;\text{if}\;LFC_{M/F,m}^{\mathrm{MUBL}}<tol_{‐}\;\text{and}\;LFC_{M/F,m}^{\mathrm{CABL}}<tol_{‐}\\ 0,\;\text{otherwise} \end{array}\right.$$

where log2 fold changes, $LFC_{M/F}^{\mathrm{CABL}}$, of the metabolite‐flow rates/fluxes of templates are computed from the reconstructed GSMNs for cancer and normal cells in advance.

The tolerances for increase or decrease are defined as $tol_{+}=\log_{2}\left(1+\varepsilon \right)$ and $tol_{‐}=\log_{2}\left(1‐\varepsilon \right)$, respectively, and ε is the percentage of flux alteration. A numerical example is provided (Doc. S1) to illustrate the computation of flux template, similarity ratio, and logarithmic fold change. The log2 fold change between the metabolite‐flow rate of the *m*th metabolite in cancer or dysregulated (denoted as MU) and normal states (denoted as BL) is computed as follows:

(5) $$LFC_{M,m}^{\mathrm{MUBL}}=\log_{2}\left(\frac{r_{m,MU}}{r_{m,BL}}\right)$$

where the metabolite‐flow rate is a pool of flux‐sum synthesis rates of the *m*th metabolite in the dysregulated or normal cells, and expressed as follows:

(6) $$r_{m}=\sum_{s\in \Omega^{c}}\left(\sum_{N_{\mathrm{ij}}>0,j}N_{\mathrm{ij}}v_{f,j}‐\sum_{N_{\mathrm{ij}}<0,j}N_{\mathrm{ij}}v_{b,j}\right),m\in \Omega^{m}$$

where *N_ij_* is the stoichiometric coefficient of the *i*th metabolite participating in the *j*th reaction; **Ω** ^*c*^ is the set of metabolites located in different compartments; and **Ω** ^*m*^ is the set of metabolites in the GSMN. The bracket in Eqn (6) indicates the synthesis rates of the *m*th metabolite at its located compartment (i.e., sum up the forward fluxes, *v_f,j_*, and backward fluxes, *v_b,j_*, of the metabolite). The log2 fold change of the forward/backward flux in dysregulated and normal states is defined as follows:

(7) $$LFC_{F,f/b}^{\mathrm{MUBL}}=\log_{2}\left(\frac{v_{f/b,MU}}{v_{f/b,BL}}\right)$$

The fold changes of the template, $LFC_{M/F,m}^{\mathrm{CABL}}$ can be obtained by applying to above definition of $LFC_{M/F,m}^{\mathrm{MUBL}}$ on a reconstructed cancer model instead of dysregulated models. Note that the templates are the flux distribution patterns for cancer and normal tissue. The templates can obtain from clinical data if they are available; otherwise, they were computed from the FBA and UFD problems without the dysregulated restrictions.

### Fitness evaluation

The TLOP in Eqn (1) is a mixed‐integer optimization problem that is NP‐hard [33]. Classical algorithms for solving bilevel optimization problems use duality theory to convert the inner‐level optimization problem into constraints in the outer‐level problem. However, duality transformation is difficult for multilevel optimization problems, such as the TLOP in this study. We applied the NHDE algorithm (Doc. S2), which has been used to solve oncogene inference problems [18, 21], to infer the oncogenes of LUAD and LUSC. The problem [Eqn (1)] consisted of the crisp objectives and fuzzy equal objective to introduce a combination of weighted‐sum and minimum decisions for evaluating the fitness, η*_D_*, which was used in the NHDE algorithm as follows:

(8) $$\eta_{D}=\left[\left(\eta_{S}+\eta_{E}\right)/2+\min\left\{\eta_{S},\eta_{E}\right\}\right]/2$$

where η*_S_* is the average similarity ratios of *SR_M_* and *SR_F_*, and the membership grade, η*_E_*, is used to measure how close the fuzzy equal objective (logarithmic fold change of the metabolite‐flow rates/fluxes) of the mutant is to the template.

**Fig. 2:**
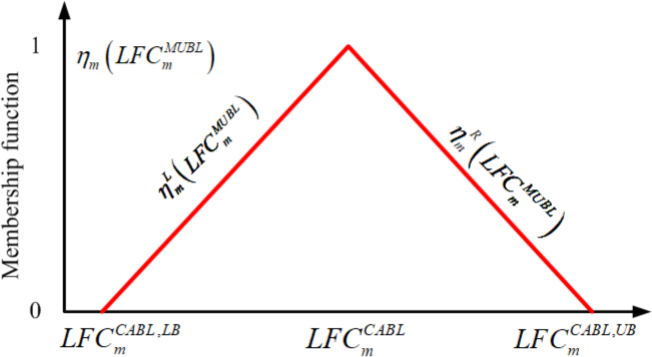
Fuzzy equal membership function. Fuzzy equal membership grade, $\eta_{m}\left(LFC_{m}^{\mathrm{MUBL}}\right)$, of a mutant consisting of a left‐hand side membership function, $\eta_{m}^{L}$, and right‐hand side membership function, $\eta_{m}^{R}$, applied to evaluate the closeness for $LFC_{m}^{\mathrm{MUBL}}$ of the mutant to the template. The membership grade is zero if $LFC_{m}^{\mathrm{MUBL}}$ exceeds the lower bound ($LFC_{m}^{CABL,LB}$) or upper bound ($LFC_{m}^{CABL,UB}$). Conversely, the membership grade is between 0 and 1 if the membership function is within its bounds.

The fuzzy equal objective for each metabolite is quantified by eliciting a membership function. In this study, the membership function is a combination of the left‐hand ($\eta_{m}^{L}$) and right‐hand ($\eta_{m}^{R}$) side linear membership functions, as shown in Fig. 2. The mathematical expressions are respectively formulated as follows:

(9) $$\eta_{m}^{L}\left(LFC_{m}^{\mathrm{MUBL}}\right)=\frac{LFC_{m}^{\mathrm{MUBL}}‐LFC_{m}^{CABL,LB}}{LFC_{m}^{\mathrm{CABL}}‐LFC_{m}^{CABL,LB}}$$

(10) $$\eta_{m}^{R}\left(LFC_{m}^{\mathrm{MUBL}}\right)=\frac{LFC_{m}^{CABL,UB}‐LFC_{m}^{\mathrm{MUBL}}}{LFC_{m}^{CABL,UB}‐LFC_{m}^{\mathrm{CABL}}}$$

where $LFC_{m}^{CABL,LB}$ and $LFC_{m}^{CABL,UB}$ are the lower and upper bounds of the log2 fold change of metabolite‐flow rate/flux in the cancer and basal states for the *m*th metabolite, and their levels can be provided by the user in advance as follows:

(11) $$LFC_{m}^{CABL,LB}=\left\{\begin{array}{l} LFC_{m}^{\mathrm{CABL}}/4,\;\text{if}\;LFC_{m}^{\mathrm{CABL}}>0\\ 4LFC_{m}^{\mathrm{CABL}},\;\text{if}\;LFC_{m}^{\mathrm{CABL}}<0 \end{array}\right.$$

(12) $$LFC_{m}^{CABL,UB}=\left\{\begin{array}{l} 4LFC_{m}^{\mathrm{CABL}},\;\text{if}\;LFC_{m}^{\mathrm{CABL}}\geq 0\\ LFC_{m}^{\mathrm{CABL}}/4,\;\text{if}\;LFC_{m}^{\mathrm{CABL}}\leq 0 \end{array}\right.$$

_The fuzzy equal membership grade for each metabolite/flux is elicited as follows:_

(13) $$\eta_{m}\left(LFC_{m}^{\mathrm{MUBL}}\right)=\max\left\{\min\left[\eta_{m}^{L}\left(LFC_{m}^{\mathrm{MUBL}}\right),\eta_{m}^{R}\left(LFC_{m}^{\mathrm{MUBL}}\right),1\right],0\right\}$$

The decision grade of the network sums the membership grades for all metabolites/fluxes as $\eta_{E}=\frac{1}{M}\sum_{m=1}^{M}\eta_{m}\left(LFC_{m}^{\mathrm{MUBL}}\right)$, which is between 0 and 1. The decision grade differs from a least‐square error criterion in regression methods. According to the definition of the fuzzy equal membership function in Eqn (13), the grade is zero if $LFC_{m}^{\mathrm{MUBL}}$ exceeds the lower bound ($LFC_{m}^{CABL,LB}$) or upper bound ($LFC_{m}^{CABL,UB}$). Conversely, the membership grade is between 0 and 1 if the membership function is within its bounds.

### Metabolite‐flow variability analysis

Generally, the optimal fluxes of FBA in the TLOP problem could have many distributions with an identical objective value. Bias in similarity ratios may be yielded through such an evaluation. To overcome such a drawback, flux variability analysis (FVA) can be applied in a posterior inspection to determine the maximum and minimum values of all fluxes that satisfy the constraints and allow for the same optimal objective value. FVA can be applied to compute minimum and maximum fluxes to yield a flux space of a metabolic network [34]. Moreover, it must cover all objective values of the cell growth because cancer cell growth may not proliferate sustainably at its maximum rate. In this study, we introduced metabolite‐flow variability analysis (MFVA) to compute the minimum and maximum quantities of each metabolite for the normal model and the mutants, respectively. The MFVA formulation was expressed as follows:

(14) $$\begin{array}{c} \text{MFVA Problem for cancer, normal, and mutant cases}\\ \left\{\begin{array}{l} \underset{\zeta \in (0,1]}{\max/\min}r_{m}\\ \text{subject to the inner optimization problems:}\\ \left\{\begin{array}{l} \text{FBA Problem:}\\ \underset{v_{f/b}}{\max}obj\equiv \left(w_{\mathrm{ATP}}v_{\mathrm{ATP}}+w_{\mathrm{biomass}}v_{\mathrm{biomass}}\right)\\ N^{CA/BL}\left(v_{f}‐v_{b}\right)=0\\ v_{f/b,j}^{\mathrm{LB}}\leq v_{f/b,j}\leq v_{f/b,j}^{\mathrm{UB}} \end{array}\right.\\ \left\{\begin{array}{l} \text{UFD problem:}\\ \min_{v_{f/b}}\left(\sum_{f}v_{f}^{2}+\sum_{b}v_{b}^{2}\right)\\ N^{CA/BL}\left(v_{f}‐v_{b}\right)=0\\ v_{f/b,i}^{\mathrm{LB}}\leq v_{f/b,i}\leq v_{f/b,i}^{\mathrm{UB}}\\ obj\geq \zeta obj^{*} \end{array}\right. \end{array}\right. \end{array}$$

The metabolite‐flow intervals, $\left[r_{m}^{\min},r_{m}^{\max}\right]$, for cancer and normal cell and each mutant can be obtained through MFVA, and were used to determine the trend of flux change between dysregulated case and normal situation in terms of seven categories of classification [21]. However, the categories were a qualitative measure to determine the trend of flux change between mutant and normal case. In this study, we introduced the interval arithmetic [35, 36] for the fuzzy equal membership grades in Eqn (13) to yield the interval membership grade as a quantitative measure to determine how much close to the template for each mutant. The interval decision grade, [η*_E_*]*_i_*, for GSMN of the *i*th mutant was calculated as follows:

(15) $${\left[\eta_{E}\right]}_{i}={\left[\eta_{E,\min},\eta_{E,\max}\right]}_{i}=\frac{1}{M}{\left[\sum_{m=1}^{M}\eta_{m,\min},\sum_{m=1}^{M}\eta_{m,\max}\right]}_{i}$$

The computational procedures of the minimum and maximum log2 fold changes (η*_E_*_,min_ and η*_E_*_,max_) are explained in detail in Doc. S3.

## Results and discussions

### Analysis of tissue‐specific metabolic models

**Fig. 3:**
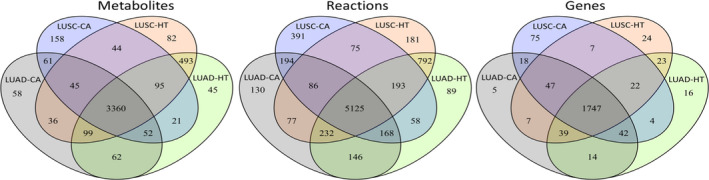
Statistics of reconstructed metabolic models. Statistics of reconstructed metabolic models for LUAD and LUSC and their corresponding healthy models. LUAD‐CA and LUSC‐CA indicate the cancer models for LUAD and LUSC, respectively, and LUAD‐HT and LUSC‐HT denote their corresponding healthy models, respectively. The number in the overlapping regions of two, three, and four models indicates their common elements.

**Fig. 4:**
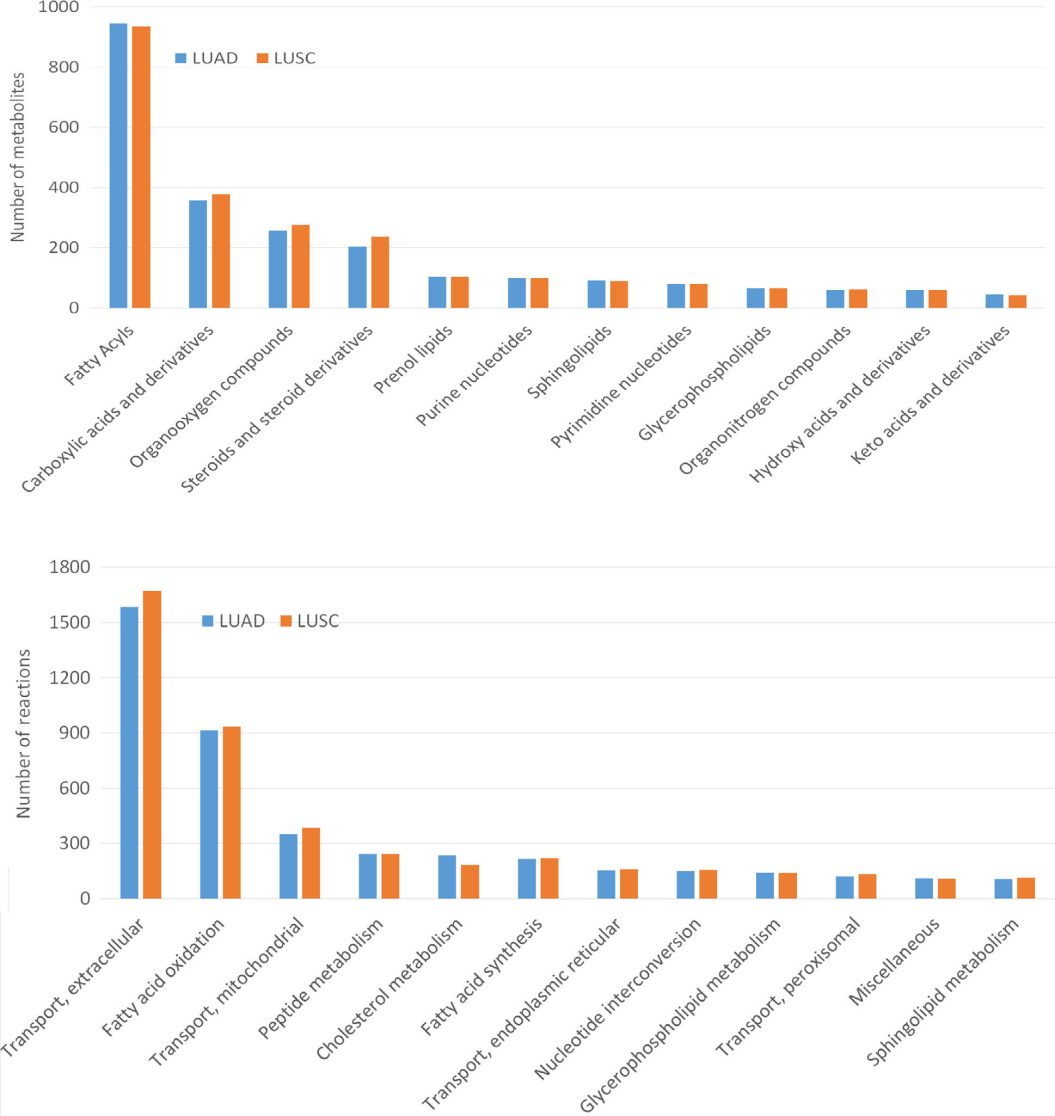
Statistics of major metabolic pathways. Classification of metabolites and reactions for the reconstructed GSMNs for LUAD and LUSC. The classifications for metabolites and reactions were sought through the definitions in the HMDB database (https://hmdb.ca/) and the VMH database (https://www.vmh.life/), respectively.

The GSMN of Recon 3D were downloaded from VHM database (https://www.vmh.life) and consisted of 5835 metabolites, 10600 reactions, and 2247 associated genes. For LUAD and LUSC, 533 and 502 cancer samples and 59 and 49 corresponding healthy samples, respectively, were obtained from TCGA database. The GSMNs for healthy and cancerous lung tissues were reconstructed using the CORDA algorithm, and statistics of the metabolites and reactions for LUAD and LUSC are presented in Fig. 3. The four models had 3360 metabolites, 5125 reactions, and 1747 genes in common, as shown in the overlapping region in Fig. 3. The cancer models comprised 3773 metabolites, 6158 reactions, and 1901 genes for LUAD and 3836 metabolites, 6290 reactions, and 1962 genes for LUSC (Fig. 3). The corresponding heathy models consisted of 4227 metabolites, 6803 reactions, and 1907 genes for LUAD and 4254 metabolites, 6761 reactions, and 1916 genes for LUSC. Twelve metabolic pathways with top‐ranked number of metabolites and reactions for both tissues are shown in Fig. 4. From the classification, we observed more than 900 and 300 metabolites for fatty acyls and carboxylic acids, respectively, for both GSMNs; more than 1500 and 900 reactions for extracellular transports and fatty acid oxidation, respectively, were observed.

Identifying differentially expressed genes (DEGs) is critical in exploring molecular mechanisms of biological conditions [37]. We assessed *P* values and fold changes (log_2_(*CA/HT*)) using ANOVA in the sas® software (https://www.sas.com/) to determine the differential expressions of enzyme‐encoding genes for LUAD and LUSC between the normal and tumor samples from TCGA database (Doc. S4). In total, 159 and 241 enzyme‐encoding genes for LUAD and LUSC, respectively, were within the absolute values of log2 fold change > 2 and *P* < 0.05, as shown in the volcano plots (Doc. S4). Such DEGs were used as a set of candidate genes to solve the oncogene inference optimization problem.

### Inferred oncogenes

**Fig. 5:**
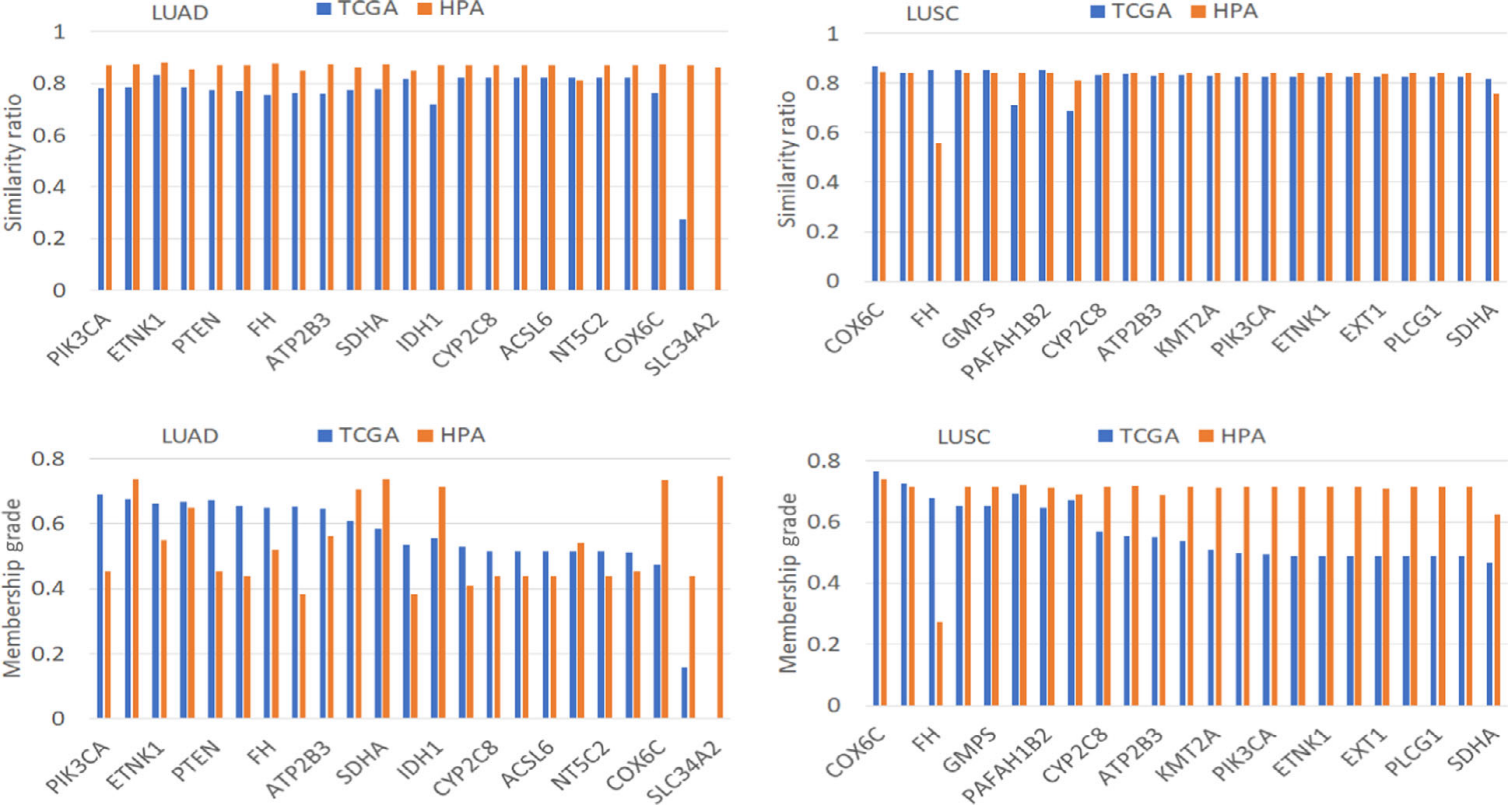
Similarity ratios and membership grades for dysregulated genes. The dysregulated genes were retrieved from the COSMIC database (https://cancer.sanger.ac.uk/cosmic). TCGA and HPA indicate the similarity ratios and membership grades computed using the GSMNs reconstructed based on data from the TCGA and HPA databases, respectively. *ATP1A1* and *CHST11* genes are not available in the GPR association of the reconstructed GSMNs.

The Catalogue Of Somatic Mutations In Cancer (COSMIC) database (https://cancer.sanger.ac.uk/cosmic) have collected 723 cancer genes that are somatically mutated and causally implicated in human cancer, including 45 enzyme‐encoding genes involved in Recon 3D. Total 25 out of the 45 enzyme‐encoding genes regulate reactions in Recon 3D according to the GPR association. To demonstrate the effectiveness of the oncogene inference optimization algorithm, the similarity ratio (η*_S_*) and membership grade (η*_E_*) of each dysregulation of the 25 enzyme‐encoding genes for LUAD and LUSC tissue‐specific GSMNs reconstructed based on data from different databases (TCGA and HPA) were computed (Fig. 5). The results show most of the similarity ratios for LUAD are greater than 0.76, except *CANT1* and *SLC34A2*, and greater than 0.8 for LUSC. However, the membership grade varies from case to case (with value ranging from 0.16 to 0.76).

The NHDE algorithm in [18, 21] was applied to evaluate the fitness in inferring carcinogenesis for all candidate enzyme‐encoding genes. High DEGs (159 genes for LUAD and 241 genes for LUSC) for both tissues were first applied individually to enable the TLOP to determine the carcinogenicity of each gene. We obtained 9 of 159 genes for LUAD and 21 of 241 genes for LUSC that have high levels of differential expression in the computation. The optimal fitness, η*_D_*, for these dysregulations were within [0.69, 0.76] for LUAD and [0.76, 0.81] for LUSC (Doc. S5). Additionally, the NHDE algorithm was also applied to identify oncogenes that have a low level of differential expression. We determined that 36 and 63 oncogenes for LUAD and LUSC had > 0.72 and > 0.76 optimal fitness, respectively (Doc. S5), in the computation.

Tables 1 and 1, 2 list the 15 top‐ranked oncogenes for LUAD (η*_D_* > 0.75) and LUSC (η*_D_* > 0.85), respectively. Pyruvate kinase (PKM) had the highest fitness (η*_D_* = 0.781), and it plays a pivotal role in regulating glucose‐derived carbon from catabolic to biosynthetic pathways. Its dysregulation is a hallmark of tumorigenesis [38, 39, 40] and leads to several cancers, such as breast cancer, renal cell carcinoma, hepatocellular carcinoma, and colorectal cancer. There are four isozymes of pyruvate kinase in mammals (L, R, M1, and M2) encoded by two different genes: *PKLR* and *PKM*. The L and R isozymes are generated from the *PKLR* by differential splicing of RNA; the M1 and M2 forms are produced from the *PKM* gene by differential splicing. The *PKLR* gene achieved the ninth highest inferred oncogene, η*_D_* = 0.744, in Table 1. The optimal fitness for the dysregulated enzymes was > 0.744, which indicated that metabolic alterations for both dysregulations were > 74% consistent with the template. We also reconstructed the tissue‐specific GSMNs of LUAD and LUSC using gene expression data from the HPA database. Such models were applied to inspect the fitness of the inferred oncogenes from TCGA database, as shown in Tables 1 and 2. In comparison with data obtained from both approaches, we could decipher that higher fitness value indicates higher possibility of tumorigenesis.

**Table 1:** Top 15 inferred oncogenes of LUAD. η*_S_^TCGA^* and η*_S_^HPA^* are the average similarity ratios for reconstructed GSMNs based on the data from the TCGA and HPA databases, respectively. η*_E_^TCGA^* and η*_E_^HPA^* are the membership grades of the fuzzy equal function for reconstructed GSMNs based on the data from the TCGA and HPA databases, respectively. Higher η*_E_* values indicate higher consistency of the flux alterations with the template. DEG and *P* value were calculated in sas® software. The pathway for each gene was found from the GeneCards (https://www.genecards.org/) and VMH (https://www.vmh.life/) databases. A gene is biological significant if |DEG| > 2 and *P* value < 0.05.

Gene	DEG^a^	*P* value^b^	(η*_S_^TCGA^*, η*_E_^TCGA^*)	(η*_S_^HPA^*_,_ η*_E_^HPA^*)	Pathway	Disease (score)^c^
*PKM*	0.98	1.01E−62	(0.842, 0.761)	(0.874, 0.734)	Abacavir pathway	Breast adenocarcinoma (0.90)
*ENO1*	1.36	4.78E−78	(0.839, 0.745)	(0.856, 0.747)	HIF‐1‐α transcription factor network	Lung cancer susceptibility 3 (0.72)
*PTDSS1*	0.17	1.29E−06	(0.835, 0.741)	(0.851, 0.725)	Glycerophospholipid biosynthetic pathway	Polyneuropathy (1.34)
*GLTP*	−0.17	6.10E−06	(0.834, 0.736)	(0.872, 0.737)	Sphingolipid metabolism	Cervical squamous cell carcinoma (0.44)
*OCRL*	0.52	3.91E−49	(0.831, 0.738)	NA	3‐phosphoinositide degradation	Lowe oculocerebrorenal syndrome (2.11)
*CA12*	1.60	5.26E−12	(0.831, 0.736)	(0.870, 0.439)	Nitrogen metabolism	Hemangioma of subcutaneous tissue (1.42), lung cancer (0.37)
*SLC22A7*	0.79	3.10E−03	(0.837, 0.728)	(0.873, 0.737)	Zidovudine pathway	renal cell carcinoma (0.64)
*PLPP1*	−0.17	3.30E−02	(0.825, 0.737)	(0.545, 0.165)	Triacylglycerol biosynthesis	Myxosarcoma (1.33)
*PKLR*	2.17	4.34E−04	(0.830, 0.715)	(0.875, 0.740)	Abacavir pathway	Intracortical osteogenic sarcoma (1.50)
*MTHFD2*	1.53	1.15E−47	(0.832, 0.727)	(0.878, 0.757)	Nucleotide metabolism	Mitochondrial complex I deficiency (0.87)
*SLC25A11*	−0.13	6.69E−03	(0.842, 0.716)	(0.868, 0.734)	Glucose metabolism	Paragangliomas 6 (2.83)
*ALDH4A1*	0.23	8.64E−04	(0.835, 0.723)	(0.858, 0.731)	Alanine, aspartate and glutamate metabolism	Hyperprolinemia, type Ii (2.48)
*GAPDH*	1.93	4.52E−61	(0.826, 0.731)	(0.855, 0.733)	Cori cycle	Angioimmunoblastic T‐cell lymphoma (1.14)
*SLC13A5*	3.24	2.64E−07	(0.835, 0.721)	(0.860, 0.731)	Transport of glucose and other sugars	Nasal cavity benign neoplasm (1.50)
*SLC20A1*	0.92	2.65E‐22	(0.834, 0.721)	(0.867, 0.736)	Glucose/energy metabolism	Leukemia (0.86)

^a^ DEG = log2(CA/HT) denotes a differential expression gene and is computed from cancer and healthy samples of TCGA datasets.

^b^ *P* value is computed from cancerous and healthy samples of TCGA datasets.

^c^ Diseases and scores are obtained from the GeneCards database.

**Table 2:** Top 15 inferred oncogenes of LUSC. η*_S_^TCGA^* and η*_S_^HPA^* are the average similarity ratios for reconstructed GSMNs based on the data from the TCGA and HPA databases, respectively. η*_E_^TCGA^* and η*_E_^HPA^* are the membership grades of the fuzzy equal function for reconstructed GSMNs based on the data from the TCGA and HPA databases, respectively. Higher η*_E_* values indicate higher consistency of the flux alterations with the template. DEG and *P* value were calculated in sas® software. The pathway for each gene was found from the GeneCards (https://www.genecards.org/) and VMH (https://www.vmh.life/) databases. A gene is biological significant if |DEG| > 2 and *P* value < 0.05.

Gene	DEG^a^	*P* value^b^	(η*_S_^TCGA^*, η*_E_^TCGA^*)	(η*_S_^HPA^*_,_ η*_E_^HPA^*)	Pathway	Disease (score)^c^
*SLCO2B1*	−2.22	8.80E−20	(0.917, 0.829)	(0.871, 0.842)	Atenolol pathway	Ileum cancer (1.31)
*SLC9A1*	−0.22	3.08E−03	(0.911, 0.822)	(0.847, 0.826)	Osteoclast signaling	Gastroesophageal reflux (1.00)
*SLC7A10*	2.68	1.14E−02	(0.911, 0.819)	(0.862, 0.835)	Differentiation of white and brown adipocyte	Follicular lymphoma (0.91)
*SLC20A1*	0.42	3.45E−04	(0.904, 0.822)	(0.870, 0.843)	Glucose/energy metabolism	Leukemia (0.86)
*AQP8*	−1.27	1.85E−05	(0.906, 0.817)	(0.878, 0.854)	Detoxification of reactive oxygen species	Colorectal adenoma (0.89)
*AGL*	0.42	1.49E−12	(0.908, 0.814)	NA	Glycogen metabolism	Bladder lateral wall cancer (1.26)
*SLC12A4*	−1.11	8.74E−40	(0.906, 0.815)	(0.871, 0.841)	Transport of glucose and other sugars	Fish‐eye disease (1.50)
*KYAT1*	0.74	2.25E−36	(0.909, 0.812)	(0.865, 0.838)	Selenocompound metabolism	Schizophrenia (0.74)
*SLC6A2*	4.35	1.46E−16	(0.897, 0.818)	NA	Methylphenidate pathway	Adrenal medulla cancer (1.21)
*SLC5A12*	4.93	1.70E−63	(0.898, 0.816)	(0.867, 0.631)	NRF2 pathway	Follicular lymphoma (0.91)
*SLC4A2*	−0.32	3.49E−07	(0.903, 0.810)	(0.867, 0.631)	Bile secretion	Hepatocellular carcinoma (0.68)
*SLCO1C1*	−0.91	1.11E−04	(0.898, 0.813)	(0.867, 0.631)	Transport of vitamins and nucleosides	Allan–Herndon–Dudley syndrome (1.20)
*SLC23A2*	−0.26	1.75E−04	(0.897, 0.814)	(0.866, 0.839)	Metabolism of water‐soluble vitamins and cofactors	Hepatitis C virus (0.87)
*ACE2*	0.77	2.46E−06	(0.896, 0.815)	(0.867, 0.631)	A‐beta plaque formation and APP metabolism	Renal oncocytoma (0.72)
*SLC43A1*	−0.97	9.28E−14	(0.897, 0.810)	(0.866, 0.840)	Amino acid transport across the plasma membrane	Seminoma (1.07)

^a^ DEG = log2(CA/HT) denotes a differential expression gene and is computed from cancer and healthy samples of TCGA datasets.

^b^ *P* value is computed from cancerous and healthy samples of TCGA datasets.

^c^ Diseases and scores are obtained from the GeneCards database.

Both PKM and PKLR isozymes are involved in the catalysis reaction (R_PYK) of phosphoenolpyruvate‐to‐pyruvate conversion. Furthermore, PKM not only catalyzes R_PYK but also the reaction of R_RE2954C, that is, the conversion of phosphoenolpyruvate and deoxythymidine‐5’‐diphosphate to pyruvate and deoxythymidine‐5'‐triphosphate. Moreover, PKLR catalyzes the R_r0280 reaction to convert phosphoenolpyruvate and deoxyadenosine diphosphate to pyruvate and deoxyadenosine triphosphate. The three aforementioned reactions used different metabolites to transform phosphoenolpyruvate to pyruvate. In addition, the products, namely deoxythymidine‐5'‐triphosphate, adenosine triphosphate, and deoxyadenosine triphosphate, of the three reactions acted as precursors of biomass reactions. We applied a reaction‐based approach discussed in the oncogene inference optimization problem [18] to determine which reaction was a dominant malfunction in LUAD and LUSC. The optimal similarity ratios (η*_S_*) and membership grades (η*_E_*) for these dysregulated reactions were similar (Table 3). *PKM* dysregulated R_PKY and R_RE2954C, which yielded a slightly higher η*_S_* and η*_E_* than did *PKLR*. Both *PKM* and *PKLR* dysregulated three reactions to obtain nearly the same characteristic. The flux fold change of each reaction was greater than twofold in pyruvate synthesis, which was consistent with the Warburg effect of enhanced pyruvate formation to increase lactate production (Table 3).

**Table 3:** Comparison of carcinogenicity caused by reaction‐based and enzyme‐based dysregulations in LUAD and LUSC, respectively. Flux fold change (LFC) is denoted as log2 fold change between cancer and healthy states; LFC was computed as log2(*r*_cancer_/*r*_normal_). η*_S_* and η*_E_* indicate the average similarity ratio and fuzzy equal membership grade, respectively. H, proton; PEP, phosphoenolpyruvate; DTDP, deoxythymidine‐5'‐diphosphate; ADP, adenosine diphosphate; DADP, deoxyadenosine diphosphate; PYR, pyruvate; ATP, adenosine triphosphate; DTTP, deoxythymidine‐5'‐triphosphate; DATP, deoxyadenosine triphosphate.

Dysregulated reactions/genes	Reactions	LUAD		LUSC	
		LFC	(η*_S_*_,_ η*_E_*)	LFC	(η*_S_*_,_ η*_E_*)
R_PYK	H + ADP + PEP → ATP + PYR	3.753	(0.829, 0.723)	3.128	(0.876, 0.754)
R_RE2954C	H + PEP + DTDP ↔ PYR + DTTP	3.671	(0.843, 0.745)	3.687	(0.867, 0.738)
R_r0280	H + PEP + DADP → PYR + DATP	3.901	(0.830, 0.714)	3.361	(0.882, 0.760)
R_RE2954C + R_r0280	H + PEP + D PYR + DTTP H + PEP + DADP → PYR + DATP	2.620 2.324	(0.841, 0.743)	2.60 2.239	(0.868, 0.740)
*PKM*	H + ADP + PEP → ATP + PYR H + PEP + DTDP ↔ PYR + DTTP	2.260 2.868	(0.842, 0.761)	1.857 2.424	(0.860, 0.728)
*PKLR*	H + ADP + PEP → ATP + PYR H + PEP + DADP → PYR + DATP	2.563 2.878	(0.830, 0.715)	2.395 2.680	(0.869, 0.752)
*PKM* + *PKLR*	H + ADP + PEP → ATP + PYR H + PEP + DTDP ↔ PYR + DTTP H + PEP + DADP → PYR + DATP	1.749 2.300 2.019	(0.848, 0.754)	1.735 2.286 1.984	(0.863, 0.717)

Enolase 1 (ENO1), along with *PKM* and *PKLR*, is a glycolytic enzyme. Glycolysis is an ATP‐generating step that is pivotal in cancer cell proliferation and metastasis. *ENO1* is overexpressed in several tumor types, including NSCLC [41, 42, 43]. ENO1 is an upstream enzyme of PKM and PKLR that catalyzes 2‐phosphoglycerate to form phosphoenolpyruvate. We observed that its DEG for LUAD obtained from RNA‐Seq datasets in TCGA was not statistically significant (DEG = 1.36). Furthermore, the computation results revealed that the flux fold change increased by 1.33 times from normal to cancer states. The average similarity ratio (η*_S_* = 0.839) and membership grade (η*_E_* = 0.745) of ENO1 were slightly smaller than those of PKM.

Phosphatidylserine synthase, encoded by *PTDSS1*, is involved in phosphatidylserine biosynthetic pathway, which is a part of phospholipid metabolism. Phosphatidylserine is a precursor in the biomass reaction of the reconstructed metabolic model. Its dysregulation causes Lenz–Majewski syndrome, which is a rare disease characterized by complex craniofacial, dental, cutaneous, and limb abnormalities combined with intellectual disability [44]. Phosphatidylserine signaling is highly dysregulated in the tumor microenvironment and autoimmune diseases [45]. According to the computation results, 1.1% upregulation of *PTDSS1* in LUAD could increase the production of phosphatidylserine that exhibited carcinogenic effects that yielded the average similarity ratio (η*_S_* = 0.835) and membership grade (η*_E_* = 0.741). By contrast, *PTDSS1* was one of the 34 top‐ranked oncogenes for LUSC that yielded η*_S_* and η*_E_* values of 0.899 and 0.789 (Doc. S5), respectively. A survival analysis for oncogenes can be applied to investigate the clinical significance of metabolic alterations. In this study, we surveyed a survival analysis from the HPA database to explain the survival significance of the inferred oncogenes. The high expression of *PTDSS1* in LUAD could significantly reduce the survival probability compared with that of PKM, PKLR, and ENO1 (Doc. S6).

Moreover, we also reconstructed LUAD and LUSC GSMNs for comparison using the iMAT algorithm [46] based on data from different databases (TCGA and HPA). The similarity ratios and membership grades for each dysregulated gene are shown in Doc. S7. The results show that the similarity ratios of LUAD and LUSC could fulfill the prediction. Because the GSMNs reconstructed by the iMAT algorithm are more parsimonious than by the CORDA algorithm, some genes are not available in the GPR association of the GSMNs.

### Dysregulation of membrane transporters

The computation results (Doc. S5) reveal that 12 out of 45 genes for LUAD and 45 out of 84 genes for LUSC served as solute carriers (SLCs). SLC genes could easily cause carcinogenesis, and we found that 11 SLC genes for LUAD (except *SLC6A4*) are common to those for LUSC. These SLC gene‐encoding proteins are categorized into SLC families and SLC anion transporter families. Such transmembrane transporters could mediate the influx and efflux of substances such as ions, nucleotides, and sugars across biological membranes. The dysregulation of these genes can drive metabolic diseases, such as type II diabetes. Reports have indicated that the mediation of *SLC5A2* and *SLC13A5* genes may be therapeutic targets for treating type II diabetes and nonalcoholic fatty liver diseases [47, 48]. In the computation, *SLC13A5* was determined to be an oncogene in LUAD and LUSC, and it had high gene differential expression (DEG = 3.239 for LUAD and 5.126 for LUSC) between cancer tissues and healthy tissues. However, *SLC5A2* was identified in LUSC only, but it possessed low gene differential expression (DEG = −1.1228). *SLC5A2* encodes a member of the sodium glucose cotransporter family, which is a sodium‐dependent glucose transport protein. The dysregulation of *SLC5A2* could lead to non‐small‐cell lung cancer and pancreatic [49] and prostate adenocarcinomas [50].

SLC13A5 is a sodium‐coupled citrate transporter that plays a key role in importing citrate from the bloodstream into human cells. Surveys conducted using PubMed and GeneCards indicated that SLC13A5 is related to nasal cavity neoplasm and hepatocellular carcinoma [51]. The computation revealed that the fold change of its mediated flux increased more than sixfold. Other common genes, namely *SLC25A11*, *SLC20A1,* and *SLC22A7*, achieved high fitness (Table 1). The oxoglutarate carrier SLC25A11 is important for ATP production in cancer during NADH transportation from the cytosol to mitochondria as a malate. The dysregulation of SLC25A11 could lead to non‐small‐cell lung cancer [52] and liver cancer [53]. Sodium‐dependent phosphate transporter 1 (SLC20A1) plays a fundamental housekeeping role in phosphate transport, such as absorbing phosphate from interstitial fluid for normal cellular functions such as cellular metabolism, signal transduction, and nucleic acid and lipid synthesis. Some review articles [54] have indicated that the phosphate transporter is overexpressed in tumor cells, which was consistent with the fold change of DEG (0.92 for LUAD and 0.42 for LUSC) evaluated from TCGA database; therefore, it has been considered a key promoter of tumorigenesis.

Angiotensin‐converting enzyme 2, encoded by *ACE2*, has recently garnered widespread interest as the SARS‐CoV‐2 receptor. It appears to be an infective agent responsible for coronavirus disease 2019 and associated cardiovascular diseases [33, 55]. We determined that *ACE2* was an oncogene of LUSC and achieved an average similarity ratio of 0.896 and membership grade of 0.815, making it one of the 15 top‐ranked oncogenes. However, *ACE2* was not implicated to cause tumorigenesis in LUAD because although its average similarity ratio could reach 0.82, its membership grade was 0.385, which was smaller than those of the 15 top‐ranked oncogenes. Furthermore, we inspected RNA‐Seq data from TCGA and found that fold changes in DEG (1.67 for LUAD and 0.77 for LUSC) for both cancers increased nonsignificantly. According to the HPA database, the high expression of *ACE2* in LUSC indicated high survival probability during the initial stages, but the results after 11 years were identical (Doc. S6). By contrast, high or low *ACE2* expression in LUAD was not differentiated.

### Results of MFVA

**Fig. 6:**
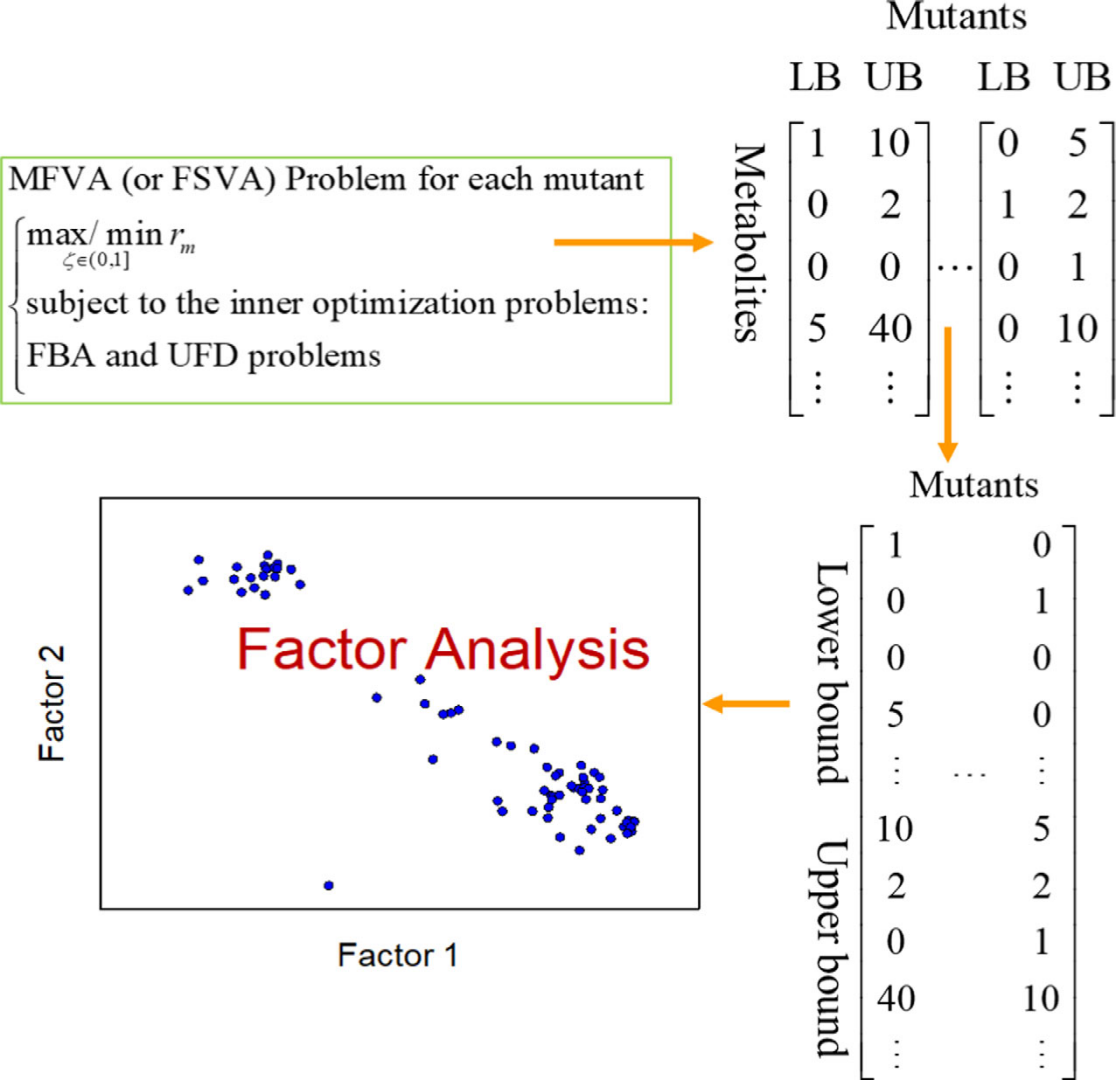
Concept of MFVA. Concept for establishing flux‐sum bounds of each mutant through MFVA for factor analysis.

**Fig. 7:**
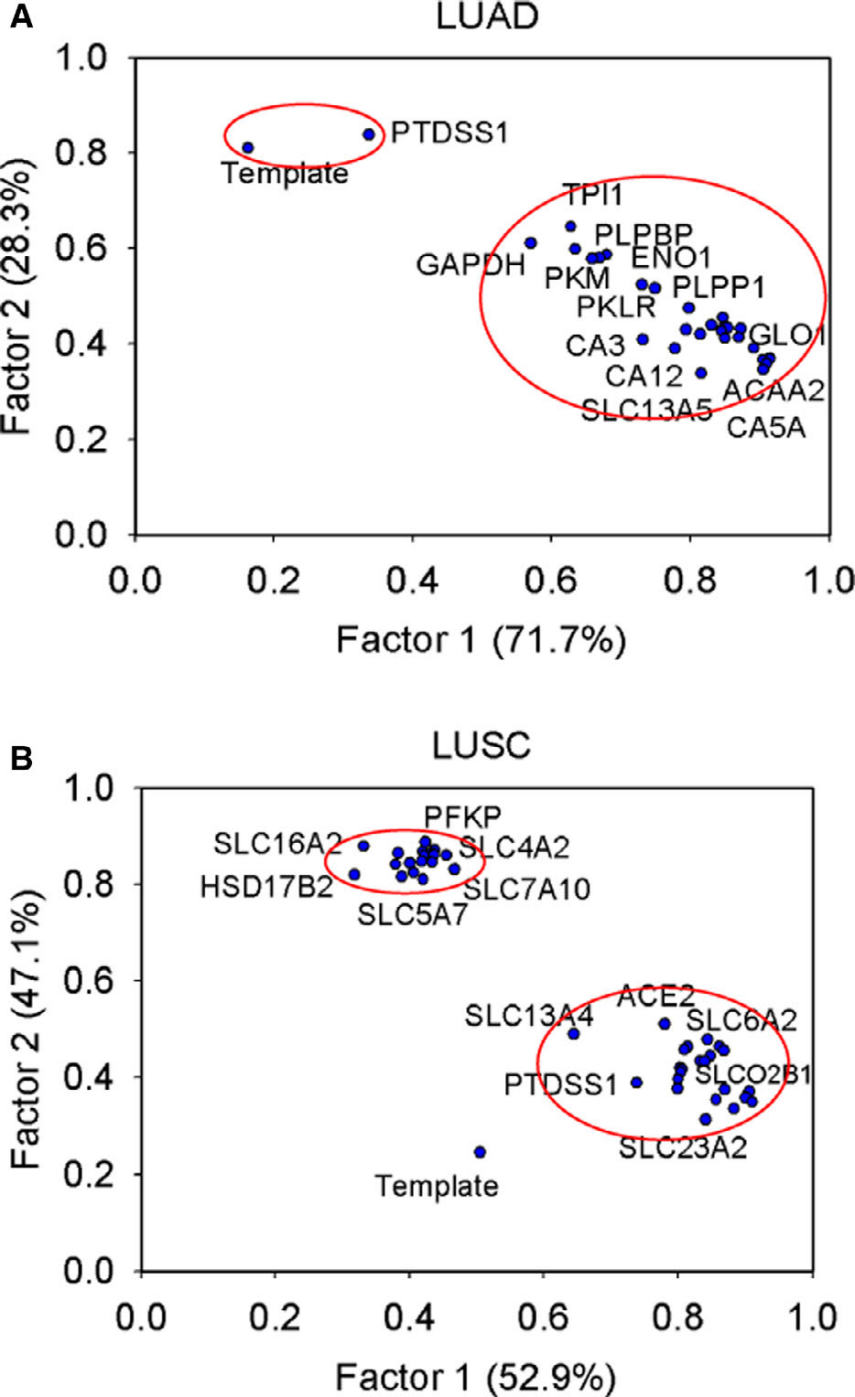
Factor analysis. Factor analysis for analyzing the flux‐bound matrices of (A) LUAD and (B) LUSC to yield two factors.

In this study, we applied the TLOP to infer oncogenes in GSMNs of lung adenocarcinoma and lung squamous cell carcinoma. FBA was involved in the inner optimization problem of TLOP. FBA can calculate steady‐state metabolic fluxes for GSMNs in a reasonable computational time with modern personal computers, but it is a biased method in constraint‐based modeling approaches for yielding optimal flux distributions. Monte Carlo sampling methods for GSMNs can cope with such a biased prediction, but still spend a lot of computer time [56]. In this study, we introduced an interval arithmetic [35, 36] for MFVA, an extension of FBA, to determine the robustness of metabolic models in various simulation conditions. However, its use has been somewhat limited by the long computation time compared with FBA. MFVA is generally incapable of embedding in the oncogene inference problem [Eqn (1)] due to the computational burden, but it could be applied to investigate whether the optimal results were achieved. MFVA was applied to calculate the lower and upper flux‐sum bounds (i.e., 4366 observed components) for each dysregulated gene. The metabolic flux‐sum bound matrix was formed by representing each dysregulated gene as a column of lower and upper flux‐sum bounds (Fig. 6) as calculated through MFVA. Moreover, the flux‐sum bound column of the template was added to the matrix. Such metabolic flux‐sum bound matrices for all mutants of LUAD and LUSC were then used to perform a factor analysis to yield two factors (Fig. 7A and B).

In total, 23 genes of LUAD in Factor 1 had factor loadings > 0.73 (Fig. 7A; numerical data are listed in Doc. S8). Acetyl‐CoA acyltransferase 2, encoded by *ACAA2*, and lactoylglutathione lyase, encoded by *GLO1*, had the two highest factor loadings (0.914) in Factor 1. ACAA2 catalyzes the final step of the mitochondrial fatty acid beta‐oxidation pathway. To date, clinical diseases caused by mutations or variants of ACAA2 have not been identified. However, the ACAA2 locus has been associated with abnormal blood lipid levels, particularly HDL and LDL cholesterol levels [55]. GLO1 participates in pyruvate metabolism to convert S‐lactoylglutathione into methylglyoxal and glutathione, and regulates TNF‐induced transcriptional activity of NF‐κB. For Factor 2, phosphatidylserine synthase 1, encoded by *PTDSS1*, had a factor loading of 0.84, which was close to the template (0.81). PTDSS1 mainly catalyzes the conversion of phosphatidylcholine in the phosphatidylserine biosynthesis pathway, which is part of phospholipid metabolism.

The factor analysis of 42 separate dysregulated genes for LUSC in two groups is shown in Fig 7B (Doc. S8). The 23 genes of Factor 1 in the first group had factor loadings > 0.64 (Fig. 7B). The template was still close to PTDSS1 (0.74) and yielded a factor loading of 0.51. The 18 genes of Factor 2 in the second group had factor loadings > 0.81 (Fig. 7B).

**Fig. 8:**
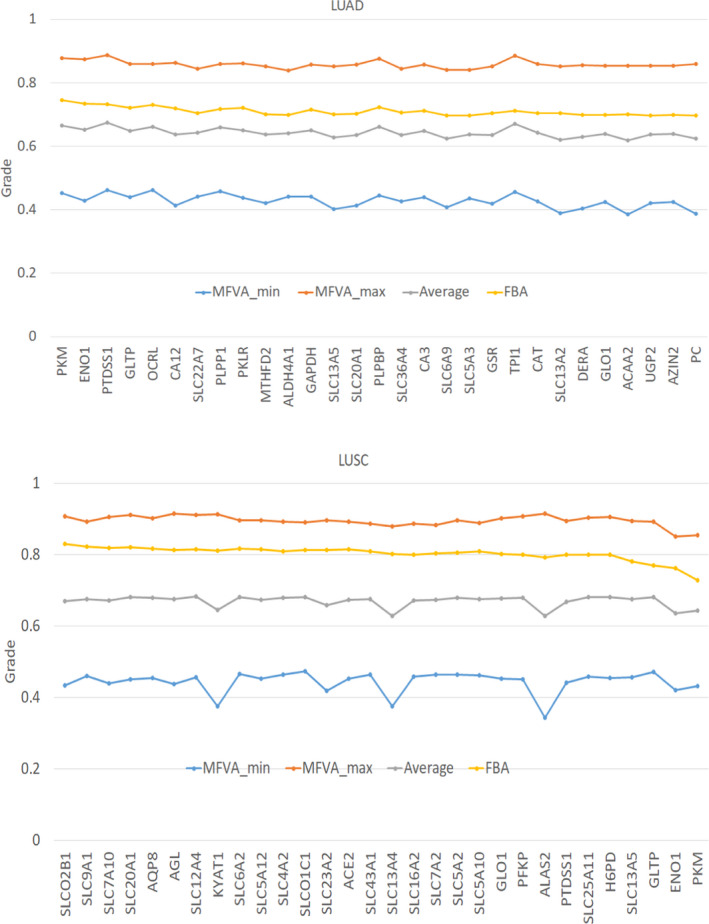
Minimum and maximum membership grades obtained through MFVA. MFVA_min and MFVA_max are the minimum and maximum fuzzy equal membership grades for each gene computed through MFVA, respectively. FBA is the optimal membership grade obtained by solving the inner optimization problem. Average is the average of MFVA_min and MFVA_max.

The lower and upper flux‐sum bounds obtained through MFVA were applied to compute the minimum and maximum membership grades (Fig. 8). In other words, the interval range of each mutant was compared with the lower and upper bounds of the template through interval computation (Doc. S9). A numerical example in Doc. S3 describes the computation of interval numbers to yield the minimum and maximum membership grades (Fig. 8). The optimal membership grade obtained through FBA in the oncogene inference problem was within the range of each mutant. PTDSS1 achieved the highest range among the mutants in LUAD, and the maximum membership grade of the mutants for LUSC was smoothly variant. According to the factor analysis, LUAD characteristics were similar to those of PTDSS1 but unobvious to those of LUSC.

## Conclusion

This study integrated the RNA‐Seq data of healthy and cancerous lung tissues downloaded from TCGA database with the genome‐scale metabolite and protein structure data of Recon 3D to reconstruct tissue‐specific GSMNs. The models were first used to generate flux patterns as a template/control in the trilevel oncogene inference optimization framework for inferring tumorigenic genes. The similarity ratios and fuzzy equal membership grades are the objectives in the oncogene inference optimization platform. The similarity ratio was used as a quality criterion to evaluate the similarity between the dysregulated flux pattern and that of the template. The fuzzy equal membership grade was used as a quantity metric to measure how close to the template the fold change of a dysregulated flux pattern is. The platform involved with the template could detect 45 and 84 tumorigenic genes for LUAD and LUSC, respectively. We observed that a high level of DEGs was not an essential factor for determining tumorigenesis. Nine out of 45 and 21 out of 84 genes for LUAD and LUSC, respectively, with high levels of differential expression based on cancer and healthy samples in TCGA database; the other genes, such as *PKM* and *PTDSS1*, have low levels of differential expression. The MFVA used the interval arithmetic to yield the interval membership grade as a quantitative measure of biased predictions from FBA.

## Conflict of interest

The authors declare no conflict of interest.

## Author contributions

FSW was responsible for study conception and design and drafted the original manuscript. WHW wrote the program and revised the manuscript. YTW, MRL, and WCC performed the data analysis and database survey. All authors have read and approved the final manuscript.

## Supporting information


**Doc. S1**. A numerical example to illustrate the computation of the template, similarity ratio, and LFC.

**Doc. S2.** Detail description of the NHDE algorithm.

**Doc. S3.** A numerical example to illustrate the interval computation of membership grade of fuzzy equal function.

**Doc. S4.** Differential expressions of enzyme‐encoding genes and volcano plots for LUAD and LUSC.

**Doc. S5.** Inferred oncogenes for LUAD and LUSC.

**Doc. S6.** Survival analysis obtained from the HPA database to explain survival significance of the inferred oncogenes.

**Doc. S7**. Similarity ratios and membership grades of the dysregulated genes from Tables 1 and 2 for the LUAD and LUSC GSMNs reconstructed by integrating the iMAT algorithm with data from different databases (TCGA or HPA).

**Doc. S8.** Results of factor analysis.

**Doc. S9.** The lower and upper flux‐sum bounds obtained through MFVA.

## Data Availability

The data that support the findings of this study are available in the Supporting information of this article.
