# Supplementary material for: Optimization of a modeling platform to predict oncogenes from genome‐scale metabolic networks of non‐small‐cell lung cancers
Source: FEBS Open Bio. 2021 Jul 20;11(8):2078–94. doi: 10.1002/2211-5463.13231 (PMC8329960; doi:10.1002/2211-5463.13231)
Supplement: Supplementary file 1 — Doc. S1. A numerical example to illustrate the computation of the template, similarity ratio, and LFC. [file FEB4-11-2078-s006.pdf]

# Numerical Example for Computing Template, Similarity Ratio and LFC

# Flowchart of the in silico experiment for inferring oncogenes

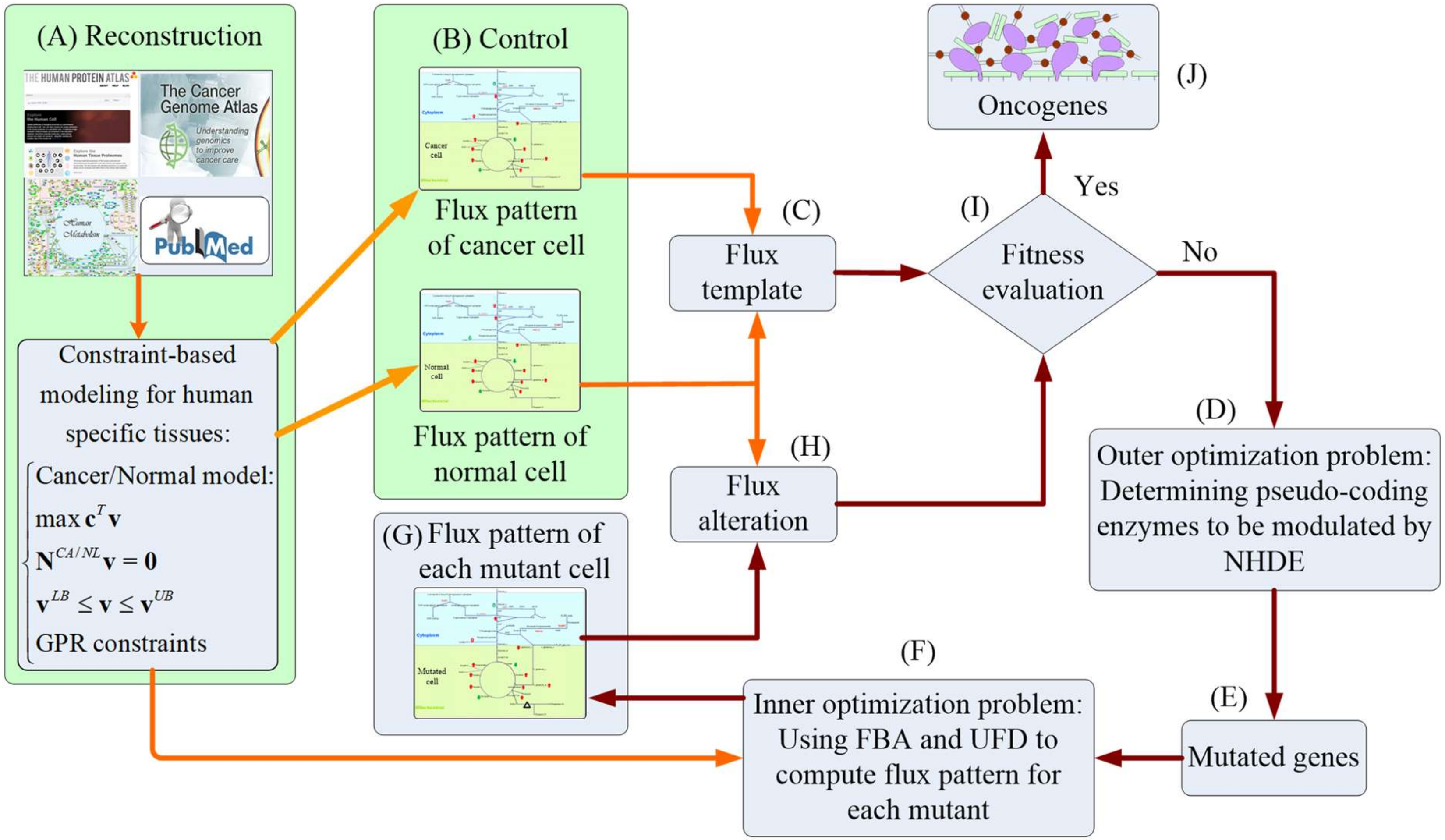


- (A) Reconstruct the cancer and normal models. (B) Compute the flux distributions of cancer and normal models. (C) Build the flux template according to the flux distributions of cancer and normal models. (D)–(J) Simulation of a wet lab experiment for determining oncogenes. Orange arrows indicate the building processes of the flux template that acted as the control in the oncogene inference problem. Red arrows present the mutant schemes for formulating the tri-level oncogene inference problem.

# Example: Template, Similarity Ratio and LFC

- We use a numerical example to illustrate the fitness evaluation in Step (D) of the above flowchart. The fitness is applied for the NHDE algorithm to infer oncogenes, iteratively.
- Suppose that a toy metabolic network consisted of 5 metabolites, and their synthesis rates for the normal model and cancer model. Both rates are then applied to build the flux template, which it serves as the control for the fitness evaluation.
- Suppose that the NHDE algorithm is selected three mutant genes, and their flux distributions as follows:

Synthesis rate for each metabolite:

$$r_m = \sum_{m_i \in \Omega^c} \left( \sum_{N_{ij}>0,j} N_{ij} v_{f,j} - \sum_{N_{ij}<0,j} N_{ij} v_{b,j} \right), m \in \Omega^m$$

| Flux Pattern | | | | | |
|---|---|---|---|---|---|
| | Normal | Cancer | Mutant 1 | Mutant 2 | Mutant 3 |
| Metabolite 1 | 1 | 4 | 2.24 | 2.1 | 3.9 |
| Metabolite 2 | 0.5 | 1.5 | 0.76 | 4 | 1.5 |
| Metabolite 3 | 1 | 0.7 | 0.93 | 0.4 | 1.07 |
| Metabolite 4 | 0.5 | 0.25 | 0.44 | 1.1 | 0.5 |
| Metabolite 5 | 1 | 1.5 | 1.04 | 1.35 | 1.48 |

- Using the above table, the log2 fold changes ($LFC_m$) for the template and each mutant are computed as follows:

| $LFC_m^T = \log_2(r_{m,\text{cancer}}/r_{m,\text{normal}})$ | | $LFC_m = \log_2(r_{m,\text{mutant}}/r_{m,\text{normal}})$ | | |
|---|---|---|---|---|
| | Template | Mutant 1 | Mutant 2 | Mutant 3 |
| Metabolite 1 | 2 | 1.1635 | 1.0704 | 1.9635 |
| Metabolite 2 | 1.585 | 0.6041 | 3 | 1.585 |
| Metabolite 3 | -0.5146 | -0.1047 | -1.3219 | 0.0976 |
| Metabolite 4 | -1 | -0.1844 | 1.1375 | 0.0 |
| Metabolite 5 | 0.585 | 0.0566 | 1.8074 | 0.5656 |

- The lower and upper bounds the template for evaluating the membership functions

Lower and upper bounds of each membership function:

$$LFC_m^{CABL,LB} = \begin{cases} LFC_m^T/4, & \text{if } LFC_m \geq 0 \\ 4 \times LFC_m^T, & \text{if } LFC_m < 0 \end{cases}$$

$$LFC_m^{CABL,UB} = \begin{cases} 4 \times LFC_m^T, & \text{if } LFC_m \geq 0 \\ LFC_m^T/4, & \text{if } LFC_m < 0 \end{cases}$$

| | $LFC_m^T\_LB$ | $LFC_m^T\_UB$ |
|---|---|---|
| Metabolite 1 | 0.5 | 8 |
| Metabolite 2 | 0.3962 | 6.3399 |
| Metabolite 3 | -2.0583 | -0.1286 |
| Metabolite 4 | -4 | -0.25 |
| Metabolite 5 | 0.1462 | 2.3399 |

- Suppose that we set 3% of the tolerance for increase/decrease, i.e. $tol_+ = 0.0426$ and $tol_- = -0.0439$
- The similarity indicator, $\mu_m$, and similarity ratio, SR are computed as follows:

Similarity indicator:

$$\mu_m^M = \begin{cases} 1, \textit{ if } LFC_M^{MUBL} > tol_+ \textit{ and } LFC_M^{CABL} > tol_+ \\ -1, \textit{ if } LFC_M^{MUBL} < tol_- \textit{ and } LFC_M^{CABL} < tol_- \\ 0, \textit{ otherwise} \end{cases}$$

| | **Similarity indicator, $\mu_m$** | | |
|---|---|---|---|
| | Mutant 1 | Mutant 2 | Mutant 3 |
| Metabolite 1 | 1 | 1 | 1 |
| Metabolite 2 | 1 | 1 | 1 |
| Metabolite 3 | -1 | -1 | 0 |
| Metabolite 4 | -1 | 0 | 0 |
| Metabolite 5 | 1 | 1 | 1 |
| $SR = \sum_{m=1}^{5} \lvert\mu_m\rvert / 5$ | 1.0 | 0.8 | 0.6 |

- The membership grades are evaluated by the left and right membership function. Both functions are defined and calculated as follows:

$$\begin{cases}\text{Left membership function:}\\ \eta_m^L(LFC_m^{MUBL}) = \dfrac{LFC_m^{MUBL} - LFC_m^{CABL,LB}}{LFC_m^{CABL} - LFC_m^{CABL,LB}} \\ \text{Right membership function:} \\ \eta_m^R(LFC_m^{MUBL}) = \dfrac{LFC_m^{CABL,UB} - LFC_m^{MUBL}}{LFC_m^{CABL,UB} - LFC_m^{CABL}} \end{cases}$$

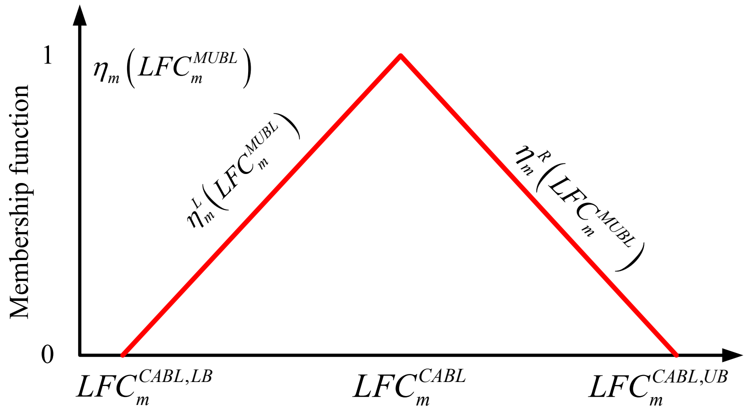


| | Mutant 1 | | Mutant 2 | | Mutant 3 | |
|---|---|---|---|---|---|---|
| Component | $\eta_m^L(LFC_m^{MUBL})$ | $\eta_m^R(LFC_m^{MUBL})$ | $\eta_m^L(LFC_m^{MUBL})$ | $\eta_m^R(LFC_m^{MUBL})$ | $\eta_m^L(LFC_m^{MUBL})$ | $\eta_m^R(LFC_m^{MUBL})$ |
| Metabolite 1 | 0.4423 | 1.1394 | 0.3803 | 1.1549 | 0.9756 | 1.0061 |
| Metabolite 2 | 0.1748 | 1.2063 | 2.1904 | 0.7024 | 1 | 1 |
| Metabolite 3 | 1.2655 | -0.062 | 0.477 | 3.092 | 1.3966 | -0.5863 |
| Metabolite 4 | 1.2719 | -0.0874 | 1.7125 | -1.85 | 1.3333 | -0.3333 |
| Metabolite 5 | -0.2044 | 1.3011 | 3.7863 | 0.3034 | 0.9559 | 1.011 |

- Using the definition of the left and right membership function, the membership grades and the fitness for each mutant are obtained as follows:

Fuzzy equal membership function:

$$\eta_m\left(LFC_m^{MUBL}\right)=\max\left\{\min\left(\eta_m^L,\eta_m^R,1\right),0\right\}$$

Mean for all metabolites:

$$\eta_E=\sum_{m=1}^{5}\eta_m/5$$

The fitness for each mutant:

$$\eta_D=\left[\left(SR+\eta_E\right)/2+\min\left\{SR,\eta_E\right\}\right]/2$$

where $SR=\eta_S$

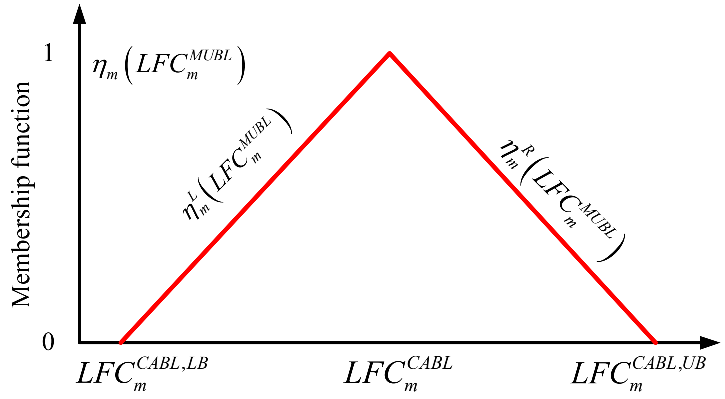


| | **Mutant 1** | **Mutant 2** | **Mutant 3** |
|---|---|---|---|
| Metabolite 1 | 0.4423 | 0.3803 | 0.9756 |
| Metabolite 2 | 0.1748 | 0.7024 | 1 |
| Metabolite 3 | 0 | 0.477 | 0 |
| Metabolite 4 | 0 | 0 | 0 |
| Metabolite 5 | 0 | 0.3034 | 0.9559 |
| $\eta_E$ | 0.1234 | 0.3726 | 0.5863 |
| $\eta_S = SR^T$ | 1.0 | 0.8 | 0.6 |
| $\eta_D$ | 0.3426 | 0.4795 | 0.5897 |

- The fitnesses for all mutants are then provided for the NHDE algorithm for generating the next mutated genes from Step (D)-(I), iteratively, towards achieving oncogenes.
