# Supplementary material for: Optimization of a modeling platform to predict oncogenes from genome‐scale metabolic networks of non‐small‐cell lung cancers
Source: FEBS Open Bio. 2021 Jul 20;11(8):2078–94. doi: 10.1002/2211-5463.13231 (PMC8329960; doi:10.1002/2211-5463.13231)
Supplement: Supplementary file 2 — Doc. S2. Detail description of the NHDE algorithm. [file FEB4-11-2078-s004.pdf]

Optimization to Predict Oncogenes in Genome-Scale Metabolic Networks of Non-Small-Cell Lung Cancers

You-Tyun Wang, Min-Ru Lin, Wei-Chen Chen, Wu-Hsiung Wu and Feng-Sheng Wang*

Department of Chemical Engineering, National Chung Cheng University, Chiayi, Taiwan

Introduction to Nested Hybrid Differential Evolution (NHDE)

The oncogene inference optimization framework is used for in silico experiments to simulate gene screening procedures and predict oncogenes. The framework is a trilevel optimization problem (TLOP), a mixed-integer optimization formulation, and described as

$$
\begin{cases}
\text{Outer optimization problem:} \\
\text{Similarity ratio of metabolite-flow rates and fluxes to the template:} \\
\quad \max\limits_{\delta, z_i} SR_M, \max\limits_{\delta, z_i} SR_F \\
\text{Fuzzy equal grade of metabolite-flow rates and fluxes} \\
\text{compared to the template:} \\
\quad \underset{\delta, z_i}{\widetilde{Equal}} LFC_M^{MUBL} \approx LFC_M^{CABL}, \underset{\delta, z_i}{\widetilde{Equal}} LFC_F^{MUBL} \approx LFC_F^{CABL} \\
\text{subject to the inner optimization problems:} \\
\begin{cases}
\text{FBA problem} \\
\max\limits_{\mathbf{v}_{f/b}} obj = \left(w_{ATP} v_{ATP} + w_{biomass} v_{biomass} \right) \\
\text{subject to} \\
\quad \mathbf{N}\left(\mathbf{v}_f - \mathbf{v}_b\right) = \mathbf{0} \\
\quad v_{f/b,i}^{LB} \le v_{f/b,i} \le v_{f/b,i}^{UB}, z_i \notin \Omega^{MU} \\
\quad v_{f/b,j}^{LB,MU} \le v_{f/b,j} \le v_{f/b,j}^{UB,MU}, z_j \in \Omega^{MU}
\end{cases}
\begin{cases}
\text{UFD problem} \\
\min\limits_{\mathbf{v}_{f/b}} \sum\limits_{i \in \Omega^{Int}} \left(v_{f,k}\right)^2 + \left(v_{b,k}\right)^2 \\
\text{subject to} \\
\quad \mathbf{N}\left(\mathbf{v}_f - \mathbf{v}_b\right) = \mathbf{0} \\
\quad v_{f/b,i}^{LB} \le v_{f/b,i} \le v_{f/b,i}^{UB}, z_i \notin \Omega^{MU} \\
\quad v_{f/b,j}^{LB,MU} \le v_{f/b,j} \le v_{f/b,j}^{UB,MU}, z_j \in \Omega^{MU} \\
\quad obj \ge obj^*
\end{cases}
\end{cases}
$$

The optimization is rewritten as the following simplified formulation for easily explaining the NHDE algorithm.

$$
\begin{cases}
\text{Outer optimization problem:} \\
\quad \max f(\mathbf{x},\mathbf{z}) \\
\text{subject to the inner optimization problems:} \\
\begin{cases}
\text{Linear programming (LP) problem} \\
\max\limits_{\mathbf{x}} obj = \mathbf{c}^T\mathbf{x} \\
\text{subject to} \\
\quad \mathbf{Ax} = \mathbf{0} \\
\quad \mathbf{x}_{LB} \le \mathbf{x} \le \mathbf{x}_{UB}, \mathbf{z} \notin \Omega^{MU} \\
\quad \mathbf{x}_{LB}^{MU} \le \mathbf{x} \le \mathbf{x}_{UB}^{MU}, \mathbf{z} \in \Omega^{MU}
\end{cases}
\begin{cases}
\text{Quadratic programming (QP) problem} \\
\min\limits_{\mathbf{x}} \mathbf{x}^T\mathbf{x} \\
\text{subject to} \\
\quad \mathbf{Ax} = \mathbf{0} \\
\quad \mathbf{x}_{LB} \le \mathbf{x} \le \mathbf{x}_{UB}, \mathbf{z} \notin \Omega^{MU} \\
\quad \mathbf{x}_{LB}^{MU} \le \mathbf{x} \le \mathbf{x}_{UB}^{MU}, \mathbf{z} \in \Omega^{MU} \\
\quad \mathbf{c}^T\mathbf{x} \ge \mathbf{c}^T\mathbf{x}^*
\end{cases}
\end{cases}
$$

The inner optimization problem consists of LP and QP problems, which is a sequential relationship.

The NHDE algorithm is a stochastic optimization based on hybrid differential evolution (HDE), which was extended from the original DE algorithm (Storn and Price, 1996; Storn and Price, 1997). The basic operations of original DE and modified NHDE are shown in Tab. S2-1. The detailed procedures have discussed by Wang (2017).

Table S2-1. Basic operations for the original DE and NHDE algorithms

**Original DE**	**Modified NHDE**
1. Representation and initialization<br>2. Mutation<br>3. Crossover operation<br>4. Selection and evaluation<br>5. Repeat steps 2 to 4	1. Representation and initialization<br>2. Mutation with rounding operation<br>3. Crossover operation<br>4. Restriction operation<br>5. Selection and evaluation<br>6. Solve LP/QP problems for each candidate gene<br>7. Compute fitness for each feasible design<br>8. Migration operation performed naturally or enforced if necessary<br>9. Repeat steps 2 to 6

The computational procedures of NHDE are listed in Tab. S2.2. NHDE is a parallel direct search algorithm (as shown in Fig.S2.1) that utilizes a population of Np individuals (enzymes) to find an optimal solution. The initialization process randomly generates Np individuals to cover the entire search space uniformly. Each individual in the population consists of a set of enzymes that are selected to be modulated.

The mutation operator of NHDE adopted from DE was an essential component compared with other evolutionary algorithms. Different from conventional evolutionary algorithms, the mutation operation of DE/NHDE uses the difference between two or four randomly chosen individuals as an evolutionary direction. The ith

mutant individual $(\mathbf{z}^G)_i$ in generation G is obtained through the difference of two or four random individuals as expressed in the following form:

$$(\mathbf{z}^G)_i = \mathrm{INT}\left\{(\mathbf{z}^G)_p + \rho^G\left[(\mathbf{z}^G)_j - (\mathbf{z}^G)_k + (\mathbf{z}^G)_l - (\mathbf{z}^G)_m\right]\right\}, i = 1,\ldots,N_p$$

where random indices $j,k,l,m \in \{1,\ldots,N_p\}$ are mutually different. The operator INT in the equation is used to rounding the real vector into the integer vector. In DE, the differential mutation factor $\rho^G \in [0, 1.2]$ is fixed and set by the user to obtain faster convergence. This factor is used to control the step length along the searching direction. A random mutation factor was used in NHDE to obtain more diversified individuals. NHDE also includes an additional mutation strategy that applying a linear crossover for the *ith* individual and the best individual $(\mathbf{z}^G)_b$ to generate the parent individual. The parent individual is therefore expressed as follows:

$$(\mathbf{z}^G)_p = \rho_p^G(\mathbf{z}^G)_b + (1-\rho_p^G)(\mathbf{z}^{G-1})_i$$

where the factor ρ_p^G is a random number between zero and one generated by a uniform distribution generator, and $(\mathbf{z}^{G-1})_i$ indicates the *ith* mutant individual in the previous generation. The mutation operation may cause the mutant individual escape from the search domain. The mutation operation may cause the mutant individual to escape the search domain (i.e., bounds are violated). If this occurs, it is replaced by a random number within the lower and upper bounds of the particular decision variable, thus restricting to the search domain. The choice of mutation factor for DE/NHDE is heuristic and random. When population diversity is low, candidate individuals rapidly cluster together such that the individuals cannot be further improved, and premature convergence occurs. Similar to conventional evolutionary algorithms, the local population diversity could be increased by using a crossover operation such as a binomial crossover.

NHDE use the difference between two or four mutually independent individuals to determine the direction of search and obtain a mutant individual. This differential mutation converges quickly so that most individuals cluster around the best candidate individual in some generations. Consequently, the population diversity and exploration capability diminish and clustered individuals are unable to reproduce more diversified individuals through the mutation operation because the weighted difference is nearly zero. The recombination of mutant individuals and their clustered parents further prevents the reproduction of a diversified population. Therefore, all individuals quickly cluster together and superior individuals cannot be generated through mutation and crossover operations.

The migration operation of the NHDE algorithm is used to help individuals escape from the local cluster, but this operation is performed only if the population diversity falls below a desired level. The degree of population diversity ζ is introduced to check whether the migration operation should be performed. Each element of the *ith*

individual ($\mathbf{z}^G$)$_i$ in generation G is referred to as a gene of the individual, and the gene diversity index dz_{ji} is given by

$$dz_{ji} = \begin{cases} 0, & \text{if } z_{ji}^G = z_{jb}^G,\ j = 1,...,n; i = 1,...,N_p; i \neq b \\ 1, & \text{otherwise,} \end{cases}$$

where z_{ji}^G and z_{jb}^G are the jth gene of the ith and best individual at the Gth generation, respectively. dz_{ji} is set to zero if the jth gene of the ith individual is identical to the best gene; otherwise it is set to one (Chiou and Wang, 1999; Liao, et al., 2001). ζ is defined as the ratio of total gene diversities to the total number of genes other than those of the best individual:

$$\zeta = \frac{\sum_{i=1, i \neq b}^{N_p} \sum_{j=1}^{n} dz_{ji}}{n(N_p - 1)}$$

The value of population diversity degree ranges between zero and one. A value of zero implies that all of the genes are clustered around the best individual. On the other hand, a value of one indicates that current candidate individuals are a completely diversified population. The desired tolerance for population diversity is assigned by the user. A tolerance value of zero implies that the migration operation in NHDE is switched off, and one implies that the migration operation is performed at every generation. Consequently, the user can set a tolerance value for population diversity degree, $\varepsilon \in (0, 1)$. If ζ is smaller than ε, then NHDE performs migration operations to regenerate a new population in order to escape from a local point; otherwise, NHDE suspends the migration operation and maintains a constant search direction toward finding a new solution.

Table S2-2. The NHDE algorithm for iteratively selecting a set of candidate enzymes and to infer optimal oncogenes

NHDE

1. Representation and initialization

$$(\mathbf{z}^0)_i = \text{uniformInt}(\mathbf{z}^{\min}, \mathbf{z}^{\max}),\, i = 1,\ldots, N_p$$

Each individual is generated by an integer random number between $\mathbf{z}_{\min}$ and $\mathbf{z}_{\max}$ with uniform distribution

2. Mutation with rounding operation

$$(\hat{\mathbf{z}}^G)_i = \text{INT}\left\{(\mathbf{z}^G)_p + \rho^G\left[(\mathbf{z}^G)_j - (\mathbf{z}^G)_k + (\mathbf{z}^G)_l - (\mathbf{z}^G)_m\right]\right\}$$

3. Crossover operation

$$z_{ji}^G = \begin{cases} z_{ji}^{G-1}, & \text{if a random number} > C_R \\ \hat{z}_{ji}^G, & \text{otherwise}, j = 1,\ldots,n;\, i = 1,\ldots, N_p \end{cases}$$

4. Restriction operation

$$z_{ji}^G = \begin{cases} z_{ji}^G, & z_{ji}^G \in [z_j^{\min}, z_j^{\max}] \\ \text{uniformInt}(z_j^{\min}, z_j^{\max}), & z_{ji}^G \notin [z_j^{\min}, z_j^{\max}] \end{cases}$$

5. Selection and evaluation
   (a) For each enzyme, solve the inner LP/QP problem by LP/QP solver, respectively
   (b) Compute fitness for each feasible solution

$$\mathit{fitness} = f(\mathbf{x}, \mathbf{z}) + \text{penalty}$$

6. Migration operation performed naturally or enforced if necessary

$$(\mathbf{z}^G)_i = \text{uniformInt}(\mathbf{z}^{\min}, \mathbf{z}^{\max}),\, \text{if } \zeta \le \varepsilon = [0,1]$$

7. Repeat steps 2 to 6

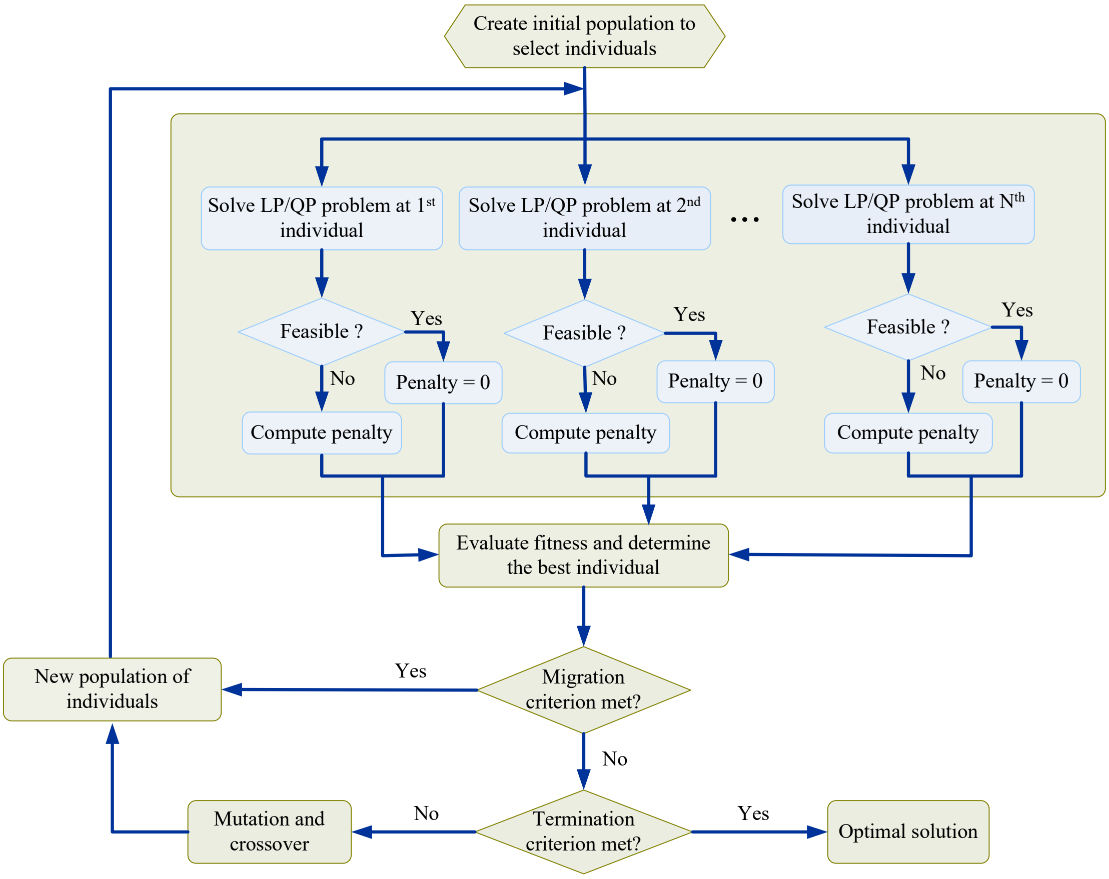

Figure S2-1. Flowchart of the parallel search algorithm in NHDE

References

Storn, R. and Price, K. (1996) Minimizing the real functions of the ICEC'96 contest by differential evolution. Evolutionary Computation, 1996., Proceedings of IEEE International Conference on. IEEE, Nagoya, pp. 842 - 844.

Storn, R. and Price, K. (1997) Differential evolution - A simple and efficient heuristic for global optimization over continuous spaces, Journal of Global Optimization, 11, 341-359.

Wang, FS (2017) Nested differential evolution for mixed-integer bi-level optimization for genome-scale metabolic networks, Ch.12, in Differential evolution in chemical engineering edited by Rangaiah and Sharma, World Scientic.

Chiou, J.P. and Wang, F.S. (1999) Hybrid method of evolutionary algorithms for static and dynamic optimization problems with application to a fed-batch fermentation process, Computers & Chemical Engineering, 23, 1277-1291.

Liao, C.T., Tzeng, W.J. and Wang, F.S. (2001) Mixed-integer hybrid differential evolution for synthesis of chemical processes, Journal of the Chinese Institute of Chemical Engineers, 32, 491-502.
