# Supplementary material for: Optimization of a modeling platform to predict oncogenes from genome‐scale metabolic networks of non‐small‐cell lung cancers
Source: FEBS Open Bio. 2021 Jul 20;11(8):2078–94. doi: 10.1002/2211-5463.13231 (PMC8329960; doi:10.1002/2211-5463.13231)
Supplement: Supplementary file 3 — Doc. S3. A numerical example to illustrate the interval computation of membership grade of fuzzy equal function. [file FEB4-11-2078-s005.pdf]

Metabolite-Flow Variability Analysis (MFVA)
or
Flux-Sum Variability Analysis (FSVA)

Roadmap for computing fuzzy equal membership grades. It is applied to measure how much closeness for the flux-sum pattern of a mutant to the template

Step (A)

FSVA Problem for Cancer or Basal Cell

$$\max/\min_{\zeta\in(0,1]} r_m$$

subject to the inner optimization problems:

FBA Problem:

$$\max_{\mathbf{v}_{f/b}} v_{biomass}$$

$$\mathbf{N}^{CA/BL}\left(\mathbf{v}_f-\mathbf{v}_b\right)=\mathbf{0}$$

$$v_{f/b,j}^{LB}\le v_{f/b,j}\le v_{f/b,j}^{UB}$$

UFD problem:

$$\min_{\mathbf{v}_{f/b}}\left(\sum_f v_f^2+\sum_b v_b^2\right)$$

$$\mathbf{N}^{CA/BL}\left(\mathbf{v}_f-\mathbf{v}_b\right)=\mathbf{0}$$

$$v_{f/b,i}^{LB}\le v_{f/b,i}\le v_{f/b,i}^{UB}$$

$$v_{biomass}\ge\zeta v_{biomass}^{*}$$

FSVA Problem for each mutant

$$\max/\min_{\zeta\in(0,1]} r_m$$

subject to the inner optimization problems:

FBA Problem:

$$\max_{\mathbf{v}_{f/b}} v_{biomass}$$

$$\mathbf{N}\left(\mathbf{v}_f-\mathbf{v}_b\right)=\mathbf{0}$$

$$v_{f/b,i}^{LB,MU}\le v_{f/b,i}\le v_{f/b,i}^{UB,MU},\ i\in\Omega^{MU}$$

$$v_{f/b,j}^{LB}\le v_{f/b,j}\le v_{f/b,j}^{UB},\ j\notin\Omega^{MU}$$

UFD problem:

$$\min_{\mathbf{v}_{f/b}}\left(\sum_f v_f^2+\sum_b v_b^2\right)$$

$$\mathbf{N}\left(\mathbf{v}_f-\mathbf{v}_b\right)=\mathbf{0}$$

$$v_{f/b,i}^{LB,MU}\le v_{f/b,i}\le v_{f/b,i}^{UB,MU},\ i\in\Omega^{MU}$$

$$v_{f/b,j}^{LB}\le v_{f/b,j}\le v_{f/b,j}^{UB},\ j\notin\Omega^{MU}$$

$$v_{biomass}\ge\zeta v_{biomass}^{*}$$

Step (B)

Minimum/Maximum flux-sum for each metabolite of cancer cell $\left[r_{m,\min}^{CA},r_{m,\max}^{CA}\right]$

Minimum/Maximum flux-sum for each metaboliteof basal cell $\left[r_{m,\min}^{BL},r_{m,\max}^{BL}\right]$

Minimum/Maximum flux-sum for each metabolite of a mutant $\left[r_{m,\min}^{MU},r_{m,\max}^{MU}\right]_i$

Step (C)

Differentiall Expression Flux-Sum of the template $\left[LFC_{m,\min}^{CABL},LFC_{m,\max}^{CABL}\right]$

Categories of Flux-Sum Intervals between Cancer/Normal Model and Mutant/Normal Model

Differentiall Expression Flux-Sum of each mutant $\left[LFC_{m,\min}^{MUBL},LFC_{m,\max}^{MUBL}\right]_i$

Step (D)

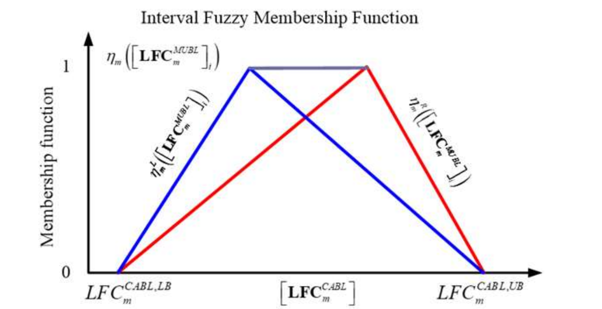

Step (E)

Fuzzy Equal Membership Grade of each mutant $\left[\eta_{m,\min},\eta_{m,\max}\right]$

Categories of Flux-Sum Intervals between Cancer/Normal Model and Mutant/Normal Model

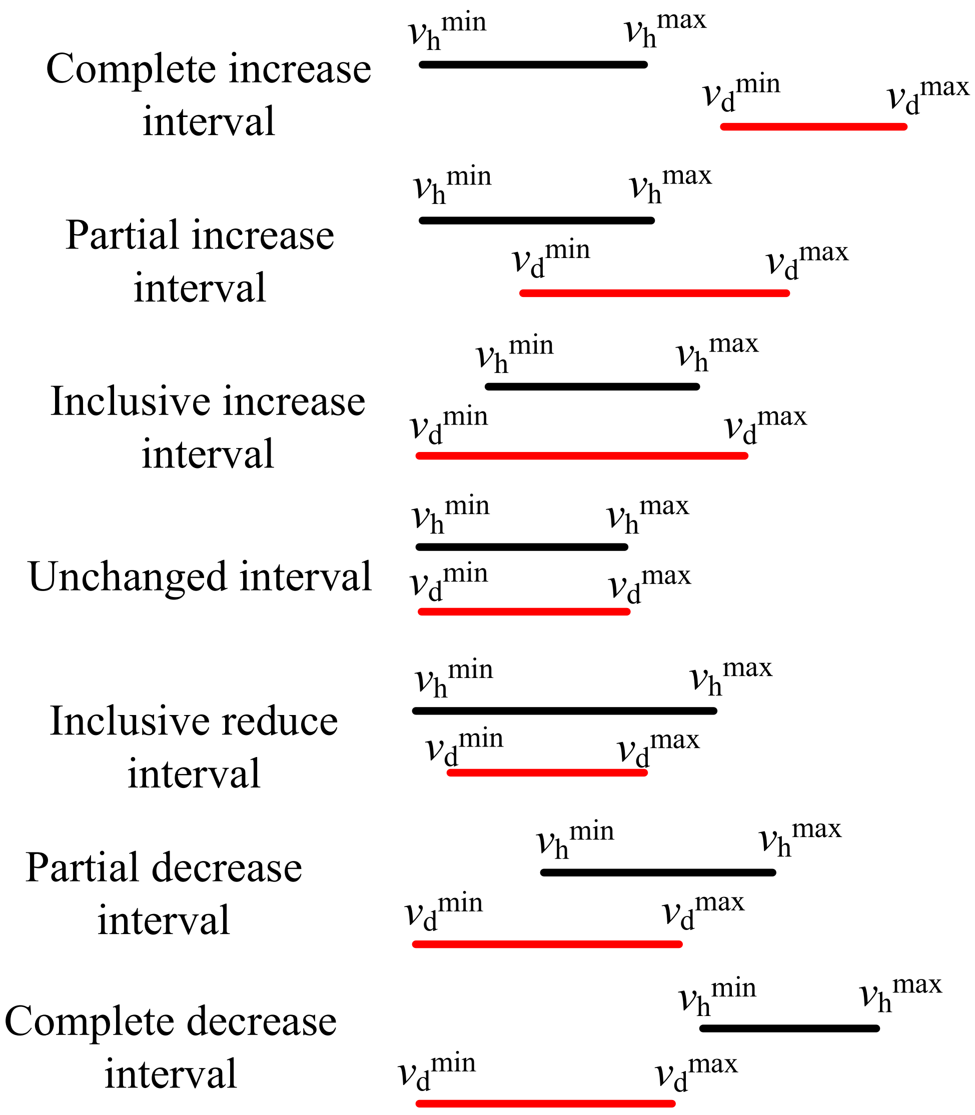

- The black line indicates the flux-sum interval of the normal model, and the red line indicates that of the mutant/cancer model.
- The categories are a qualitative measure to determine the trend of flux change between mutant and normal case, not quantitative.

Computational Procedures of Fuzzy Equal Membership Grade

- The FSVA problems for the cancer model and basal model in Step (A) are respectively applied to compute the minimum and maximum synthesis rate for each metabolite. Such interval flux-sum are expressed as shown in Step (B):

Minimum/Maximum flux-sum for each metabolite of cancer cell:

$$\left[r_{m,\min}^{CA}, r_{m,\max}^{CA}\right]$$

Minimum/Maximum flux-sum for each metaboliteof basal cell:

$$\left[r_{m,\min}^{BL}, r_{m,\max}^{BL}\right]$$

Minimum/Maximum flux-sum for each metabolite of the ith mutant:

$$\left[r_{m,\min}^{MU}, r_{m,\max}^{MU}\right]_i$$

- Using interval arithmetic, log2 interval fold changes of the template and mutants are computed as shown in Step (C):

Differentiall Expression Flux-Sum of the template:

$$\left[\mathbf{LFC}_m^{CABL}\right] = \left[LFC_{m,\min}^{CABL}, LFC_{m,\max}^{CABL}\right]$$

Differentiall Expression Flux-Sum of each mutant:

$$\left[\mathbf{LFC}_m^{MUBL}\right]_i = \left[LFC_{m,\min}^{MUBL}, LFC_{m,\max}^{MUBL}\right]_i$$

- The computation and definition of the interval arithmetic are expressed as follows:

Computation of log2 interval fold changes of the template and mutants

- From Step (B), we have the interval flux-sum of each metabolite for cancer, basal and each mutant

Minimum/Maximum flux-sum for each metabolite of cancer cell:

$$\left[r^{CA}_{m,\min}, r^{CA}_{m,\max} \right]$$

Minimum/Maximum flux-sum for each metaboliteof basal cell:

$$\left[r^{BL}_{m,\min}, r^{BL}_{m,\max} \right]$$

Minimum/Maximum flux-sum for each metabolite of the ith mutant:

$$\left[r^{MU}_{m,\min}, r^{MU}_{m,\max} \right]_i$$

- The interval fold changes of the template and each mutants are computed by interval arithmetic as

The interval fold change of the template:

$$\left[CABL_{m,\min}, CABL_{m,\max} \right] = \left[\min\left\{ \frac{r^{CA}_{m,\min}}{r^{BL}_{m,\min}}, \frac{r^{CA}_{m,\min}}{r^{BL}_{m,\max}}, \frac{r^{CA}_{m,\max}}{r^{BL}_{m,\min}}, \frac{r^{CA}_{m,\max}}{r^{BL}_{m,\max}} \right\}, \max\left\{ \frac{r^{CA}_{m,\min}}{r^{BL}_{m,\min}}, \frac{r^{CA}_{m,\min}}{r^{BL}_{m,\max}}, \frac{r^{CA}_{m,\max}}{r^{BL}_{m,\min}}, \frac{r^{CA}_{m,\max}}{r^{BL}_{m,\max}} \right\} \right]$$

The interval fold change of the ith mutant:

$$\left[MUBL_{m,\min}, MUBL_{m,\max} \right]_i = \left[\min\left\{ \frac{r^{MU}_{m,\min}}{r^{BL}_{m,\min}}, \frac{r^{MU}_{m,\min}}{r^{BL}_{m,\max}}, \frac{r^{MU}_{m,\max}}{r^{BL}_{m,\min}}, \frac{r^{MU}_{m,\max}}{r^{BL}_{m,\max}} \right\}, \max\left\{ \frac{r^{MU}_{m,\min}}{r^{BL}_{m,\min}}, \frac{r^{MU}_{m,\min}}{r^{BL}_{m,\max}}, \frac{r^{MU}_{m,\max}}{r^{BL}_{m,\min}}, \frac{r^{MU}_{m,\max}}{r^{BL}_{m,\max}} \right\} \right]_i$$

Computation of log2 interval fold changes of the template and mutants

- The log2 interval fold changes of the template and each mutant are obtained, respectively, as

Template:

$$\left[\mathbf{LFC}_m^{CABL}\right]=\left[LFC_{m,\min}^{CABL},LFC_{m,\max}^{CABL}\right]=\log 2\left(\left[CABL_{m,\min},CABL_{m,\max}\right]\right)=\left[\log 2\left(CABL_{m,\min}\right),\log 2\left(CABL_{m,\max}\right)\right]$$

ith mutant:

$$\left[\mathbf{LFC}_m^{MUBL}\right]_i=\left[LFC_{m,\min}^{MUBL},LFC_{m,\max}^{MUBL}\right]_i=\log 2\left(\left[MUBL_{m,\min},MUBL_{m,\max}\right]_i\right)=\left[\log 2\left(MUBL_{m,\min}\right),\log 2\left(MUBL_{m,\max}\right)\right]_i$$

- Define the interval fuzzy membership function to be applied as a quantitative measure to determine how closeness of the mutant interval fold changes to the template.

The user provides the lower and upper bounds of the left and right membership function:

$$LFC_{m,LB}=\begin{cases}LFC_{m,\min}^{CABL}/\delta, & LFC_{m,\min}^{CABL}\ge 0\\ \delta LFC_{m,\min}^{CABL}, & LFC_{m,\min}^{CABL}<0\end{cases}$$

$$LFC_{m,UB}=\begin{cases}LFC_{m,\max}^{CABL}/\delta, & LFC_{m,\max}^{CABL}\le 0\\ \delta LFC_{m,\max}^{CABL}, & LFC_{m,\max}^{CABL}>0\end{cases}$$

where $\delta=4$

Interval Fuzzy Membership Function

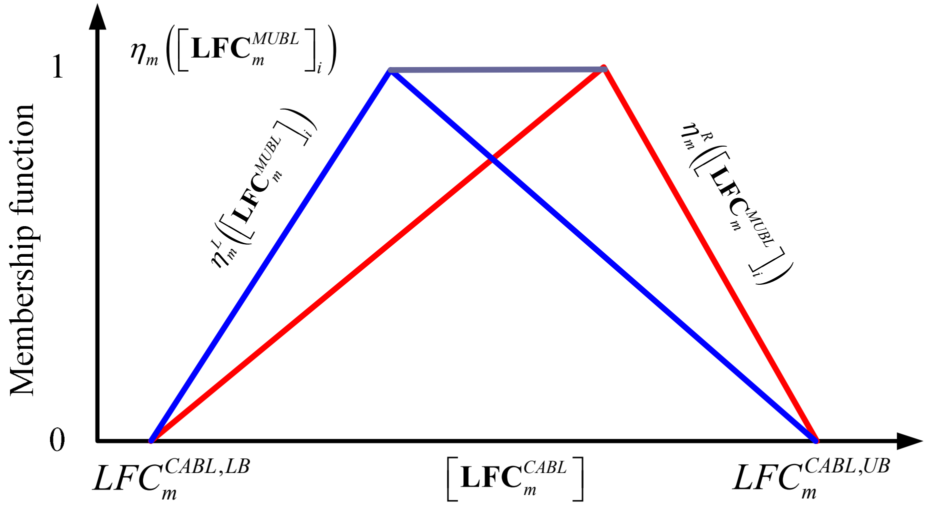

Evaluation of Interval Fuzzy Equal Membership Grade for each Mutant to the Template

- The interval left and right membership functions are defined as follows:

Interval left membership function, $\left[\boldsymbol{\eta}_m^L\right]_i$:

$$\left[\boldsymbol{\eta}_m^L\right]_i = \left[\eta_{m,\min}^L, \eta_{m,\max}^L\right]_i = \frac{\left[\mathbf{LFC}_m^{MUBL}\right]_i - LFC_{m,LB}}{\left[\mathbf{LFC}_m^{CABL}\right] - LFC_{m,LB}}$$

Interval right membership function, $\left[\boldsymbol{\eta}_m^R\right]_i$:

$$\left[\boldsymbol{\eta}_m^R\right]_i = \left[\eta_{m,\min}^R, \eta_{m,\max}^R\right]_i = \frac{LFC_{m,UB} - \left[\mathbf{LFC}_m^{MUBL}\right]_i}{LFC_{m,UB} - \left[\mathbf{LFC}_m^{CABL}\right]}$$

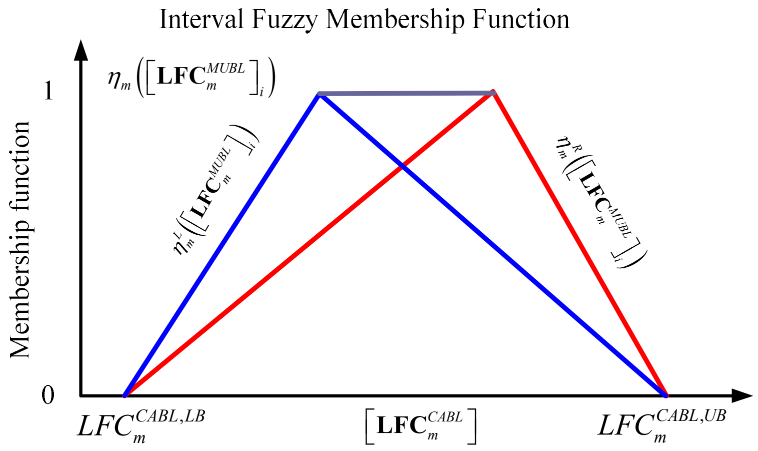

- The minimum and maximum values of interval left and right membership functions are computed by interval arithmetic as follows:

$$\left[\eta_{m,\min}^L, \eta_{m,\max}^L\right]_i = \begin{bmatrix} \min\left\{ \frac{\left(LFC_{m,\min}^{MUBL} - LFC_{m,LB}\right)}{\left(LFC_{m,\min}^{CABL} - LFC_{m,LB}\right)}, \frac{\left(LFC_{m,\min}^{MUBL} - LFC_{m,LB}\right)}{\left(LFC_{m,\max}^{CABL} - LFC_{m,LB}\right)}, \frac{\left(LFC_{m,\max}^{MUBL} - LFC_{m,LB}\right)}{\left(LFC_{m,\min}^{CABL} - LFC_{m,LB}\right)}, \frac{\left(LFC_{m,\max}^{MUBL} - LFC_{m,LB}\right)}{\left(LFC_{m,\max}^{CABL} - LFC_{m,LB}\right)} \right\}, \\ \max\left\{ \frac{\left(LFC_{m,\min}^{MUBL} - LFC_{m,LB}\right)}{\left(LFC_{m,\min}^{CABL} - LFC_{m,LB}\right)}, \frac{\left(LFC_{m,\min}^{MUBL} - LFC_{m,LB}\right)}{\left(LFC_{m,\max}^{CABL} - LFC_{m,LB}\right)}, \frac{\left(LFC_{m,\max}^{MUBL} - LFC_{m,LB}\right)}{\left(LFC_{m,\min}^{CABL} - LFC_{m,LB}\right)}, \frac{\left(LFC_{m,\max}^{MUBL} - LFC_{m,LB}\right)}{\left(LFC_{m,\max}^{CABL} - LFC_{m,LB}\right)} \right\} \end{bmatrix}$$

$$\left[\eta_{m,\min}^R, \eta_{m,\max}^R\right] = \begin{bmatrix} \min\left\{ \frac{\left(LFC_{m,UB} - LFC_{m,\min}^{MUBL}\right)}{\left(LFC_{m,UB} - LFC_{m,\min}^{CABL}\right)}, \frac{\left(LFC_{m,UB} - LFC_{m,\min}^{MUBL}\right)}{\left(LFC_{m,UB} - LFC_{m,\max}^{CABL}\right)}, \frac{\left(LFC_{m,UB} - LFC_{m,\max}^{MUBL}\right)}{\left(LFC_{m,UB} - LFC_{m,\min}^{CABL}\right)}, \frac{\left(LFC_{m,UB} - LFC_{m,\max}^{MUBL}\right)}{\left(LFC_{m,UB} - LFC_{m,\max}^{CABL}\right)} \right\}, \\ \max\left\{ \frac{\left(LFC_{m,UB} - LFC_{m,\min}^{MUBL}\right)}{\left(LFC_{m,UB} - LFC_{m,\min}^{CABL}\right)}, \frac{\left(LFC_{m,UB} - LFC_{m,\min}^{MUBL}\right)}{\left(LFC_{m,UB} - LFC_{m,\max}^{CABL}\right)}, \frac{\left(LFC_{m,UB} - LFC_{m,\max}^{MUBL}\right)}{\left(LFC_{m,UB} - LFC_{m,\min}^{CABL}\right)}, \frac{\left(LFC_{m,UB} - LFC_{m,\max}^{MUBL}\right)}{\left(LFC_{m,UB} - LFC_{m,\max}^{CABL}\right)} \right\} \end{bmatrix}$$

Evaluation of Interval Fuzzy Equal Membership Grade for each Mutant to the Template

- The interval membership grade for each metabolite of the ith mutant is defined as

$$\begin{aligned}
[\boldsymbol{\eta}_m]_i &= \left[\max\left\{ \min\left\{ \left([\boldsymbol{\eta}_m^L], [\boldsymbol{\eta}_m^R], 1 \right) \right\}, 0 \right\} \right] \\
&= \left[\eta_{m,\min}, \eta_{m,\max} \right] \\
&= \begin{bmatrix} \max\left\{ \min\left(\eta_{m,\min}^L, \eta_{m,\min}^R, 1 \right), 0 \right\}, \\ \max\left\{ \min\left(\eta_{m,\max}^L, \eta_{m,\max}^R, 1 \right), 0 \right\} \end{bmatrix}
\end{aligned}$$

- The interval decision grade for the genome-scale metabolic net of the ith mutant is defined as

$$[\boldsymbol{\eta}_E]_i = \left[\eta_{E,\min}, \eta_{E,\max} \right]_i = \left[\frac{1}{M} \sum_{m=1}^{M} \eta_{m,\min}, \frac{1}{M} \sum_{m=1}^{M} \eta_{m,\max} \right]_i$$

- The interval decision grade can be applied as a quantitative measure to determine how much closeness of each mutant to the template.
- The center and radius of the interval decision grade are defined as

$$Center = \frac{\eta_{E,\min} + \eta_{E,\max}}{2}$$

$$Radius = \frac{\eta_{E,\max} - \eta_{E,\min}}{2}$$

Definition of Computation of Interval Numbers

- Suppose that two intervals, [C] = [C_{min},C_{max}] and [B] = [B_{min}, B_{max}]

- Subtraction: $$[C]-[B]=[C_{\min}-B_{\max},C_{\max}-B_{\min}]$$

- Addition: $$[C]+[B]=[C_{\min}+B_{\min},C_{\max}+B_{\max}]$$

- Multiplication: $$[C]*[B]=\begin{bmatrix}\min\{C_{\min}B_{\min},C_{\min}B_{\max},C_{\max}B_{\min},C_{\max}B_{\max}\}, \\ \max\{C_{\min}B_{\min},C_{\min}B_{\max},C_{\max}B_{\min},C_{\max}B_{\max}\}\end{bmatrix}$$

- Division: $$[C]/[B]=\begin{bmatrix}\min\{C_{\min}/B_{\min},C_{\min}/B_{\max},C_{\max}/B_{\min},C_{\max}/B_{\max}\}, \\ \max\{C_{\min}/B_{\min},C_{\min}/B_{\max},C_{\max}/B_{\min},C_{\max}/B_{\max}\}\end{bmatrix},0\notin[B]$$

1. Arnold Kaufmann and Madan M. Guta, Introduction to Fuzzy Arithmetic: Theory and Application, Van Nostrand Reinhold,1991
2. Eduardo Massad, Laécio Carvalho de Barr, Neli Regina Siqueira Ortega and Claudio José Struchiner, Fuzzy Logic in Action: Applications in Epidemiology and Beyond, Springer, 2008
3. Hang Zettervall, Fuzzy set theory applied to make medical prognoses for cancer patients, Doctoral Dissertation, Blekinge Institute of Technology, Sweden, 2014

Example: Evaluation of Interval Membership Grades

- Suppose that a toy metabolic network consisted of 7 metabolites, and their synthesis rates for the normal model, cancer model and two mutant models are computed by FSVA

Flux Pattern by FSVA

	Basal		Cancer		Mutant 1		Mutant 2	
	BL_{min}	BL_{max}	CA_{min}	CA_{max}	$MU_{1,min}$	$MU_{1,max}$	$MU_{2,min}$	$MU_{2,max}$
Metabolite 1	0.5	1	1.5	3	1.2	2.4	1	1.5
Metabolite 2	0.2	1.2	0.5	2	0.4	2.2	0.2	2
Metabolite 3	0.4	0.8	0.4	1.5	0.4	1.2	0.4	1
Metabolite 4	0.5	0.9	0.5	0.8	0.5	0.9	0.4	0.8
Metabolite 5	0.4	0.8	0.2	0.8	0.1	0.8	0.5	1
Metabolite 6	0.2	1.2	0.1	1	0.1	1.1	0.5	1.2
Metabolite 7	1	2	0.2	0.8	0.2	0.8	1	2

- Numerical example for log2 interval fold changes of the template and mutants:

Template:

$$\left[\mathbf{LFC}_m^{CABL}\right] = \left[LFC_{m,\min}^{CABL}, LFC_{m,\max}^{CABL}\right] = \log 2\left(\left[CABL_{m,\min}, CABL_{m,\max}\right]\right) = \left[\log 2\left(CABL_{m,\min}\right), \log 2\left(CABL_{m,\max}\right)\right]$$

ith mutant:

$$\left[\mathbf{LFC}_m^{MUBL}\right]_i = \left[LFC_{m,\min}^{MUBL}, LFC_{m,\max}^{MUBL}\right]_i = \log 2\left(\left[MUBL_{m,\min}, MUBL_{m,\max}\right]_i\right) = \left[\log 2\left(MUBL_{m,\min}\right), \log 2\left(MUBL_{m,\max}\right)\right]_i$$

	Template		Mutant 1		Mutant 2	
	$LFC_{m,\min}^{CABL}$	$LFC_{m,\max}^{CABL}$	$LFC_{m,\min}^{MUBL}$	$LFC_{m,\max}^{MUBL}$	$LFC_{m,\min}^{MUBL}$	$LFC_{m,\max}^{MUBL}$
Metabolite 1	0.585	2.585	0.263	2.263	0	1.585
Metabolite 2	-1.263	3.3219	-1.585	3.4594	-2.585	3.3219
Metabolite 3	-1	1.9069	-1	1.585	-1	1.3219
Metabolite 4	-0.848	0.6781	-0.848	0.848	-1.1699	0.6781
Metabolite 5	-2	1	-3	1	-0.6781	1.3219
Metabolite 6	-3.585	2.3219	-3.585	2.4594	-1.263	2.585
Metabolite 7	-3.3219	-0.3219	-3.3219	-0.3219	-1	1

The lower and upper bound of membership functions

The user provides the lower and upper bounds of the left and right membership function:

$$LFC_{m,LB} = \begin{cases} LFC_{m,\min}^{CABL} / \delta, LFC_{m,\min}^{CABL} \geq 0 \\ \delta LFC_{m,\min}^{CABL}, LFC_{m,\min}^{CABL} < 0 \end{cases}$$

$$LFC_{m,UB} = \begin{cases} LFC_{m,\max}^{CABL} / \delta, LFC_{m,\max}^{CABL} \leq 0 \\ \delta LFC_{m,\max}^{CABL}, LFC_{m,\max}^{CABL} > 0 \end{cases}$$

where $\delta = 2$

	$LFC_{m,LB}$	$LFC_{m,\min}^{CABL}$	$LFC_{m,\max}^{CABL}$	$LFC_{m,UB}$
Metabolite 1	0.293	0.585	2.585	5.17
Metabolite 2	-2.526	-1.263	3.322	6.644
Metabolite 3	-2	-1	1.907	3.814
Metabolite 4	-1.696	-0.848	0.848	1.696
Metabolite 5	-4	-2	1	2
Metabolite 6	-13.814	-6.907	2.322	4.644
Metabolite 7	-6.644	-3.322	-0.322	-0.161

- Numerical example for interval membership function values of mutants:

Interval left membership function, $\left[\boldsymbol{\eta}_m^L\right]_i$:

$$\left[\boldsymbol{\eta}_m^L\right]_i = \left[\eta_{m,\min}^L, \eta_{m,\max}^L\right]_i = \frac{[\mathbf{LFC}_m^{MUBL}]_i - LFC_{m,LB}}{[\mathbf{LFC}_m^{CABL}] - LFC_{m,LB}}$$

Interval right membership function, $\left[\boldsymbol{\eta}_m^R\right]_i$:

$$\left[\boldsymbol{\eta}_m^R\right]_i = \left[\eta_{m,\min}^R, \eta_{m,\max}^R\right]_i = \frac{LFC_{m,UB} - [\mathbf{LFC}_m^{MUBL}]_i}{LFC_{m,UB} - [\mathbf{LFC}_m^{CABL}]}$$

	Mutant 1				Mutant 2			
	$\eta_{m,\min}^L$	$\eta_{m,\max}^L$	$\eta_{m,\min}^R$	$\eta_{m,\max}^R$	$\eta_{m,\min}^L$	$\eta_{m,\max}^L$	$\eta_{m,\min}^R$	$\eta_{m,\max}^R$
Metabolite 1	-0.1007	6.7374	0.634	1.8982	-1	4.419	0.7819	2
Metabolite 2	0.1609	4.739	0.4027	2.4771	-0.0466	4.6301	0.4201	2.7782
Metabolite 3	0.256	3.585	0.463	2.5244	0.256	3.3219	0.5176	2.5244
Metabolite 4	0.3572	3	0.2305	3.2506	0.2216	2.7996	0.3076	3.7254
Metabolite 5	0.2	2.5	0.25	5	0.6644	2.661	0.1695	2.6781
Metabolite 6	0.3777	2.686	0.2655	3.544	0.6223	2.7211	0.2502	2.544
Metabolite 7	0.5255	1.9031	0.0509	19.6377	0.8927	2.301	-7.2126	5.2126

- The interval membership grade for each metabolite

$$[\boldsymbol{\eta}_{mn}]_i = \left[\max\left\{ \min\left\{ \left([\boldsymbol{\eta}_m^L], [\boldsymbol{\eta}_m^R], 1 \right) \right\}, 0 \right\} \right]$$
$$= \left[\eta_{m,\min}, \eta_{m,\max} \right]$$
$$= \begin{bmatrix} \max\left\{ \min\left(\eta_{m,\min}^L, \eta_{m,\min}^R, 1 \right), 0 \right\}, \\ \max\left\{ \min\left(\eta_{m,\max}^L, \eta_{m,\max}^R, 1 \right), 0 \right\} \end{bmatrix}$$

$$[\boldsymbol{\eta}_E]_i = \left[\eta_{E,\min}, \eta_{E,\max} \right]_i$$
$$= \left[\frac{1}{M} \sum_{m=1}^{M} \eta_{m,\min}, \frac{1}{M} \sum_{m=1}^{M} \eta_{m,\max} \right]_i$$
$$Center = \frac{\eta_{E,\min} + \eta_{E,\max}}{2}$$
$$Radius = \frac{\eta_{E,\max} - \eta_{E,\min}}{2}$$

	**Mutant 1**		**Mutant 2**	
	$\eta_{m,min}$	$\eta_{m,max}$	$\eta_{m,min}$	$\eta_{m,max}$
Metabolite 1	0	1	0	1
Metabolite 2	0.1609	1	0	1
Metabolite 3	0.256	1	0.256	1
Metabolite 4	0.2305	1	0.2216	1
Metabolite 5	0.2	1	0.1695	1
Metabolite 6	0.2655	1	0.2502	1
Metabolite 7	0.0509	1	0	1
$[\eta_E]_i$	0.1663	1	0.1282	1
Center	0.5831		0.5641	
Radius	0.4169		0.4359	
