# Supplementary material for: Optimization of a modeling platform to predict oncogenes from genome‐scale metabolic networks of non‐small‐cell lung cancers
Source: FEBS Open Bio. 2021 Jul 20;11(8):2078–94. doi: 10.1002/2211-5463.13231 (PMC8329960; doi:10.1002/2211-5463.13231)
Supplement: Supplementary file 6 — Doc. S6. Survival analysis obtained from the HPA database to explain survival significance of the inferred oncogenes. [file FEB4-11-2078-s002.pdf]

Figure S6. Survival analysis obtained from the HPA database to explain survival significance of the inferred oncogenes discussed in Table 1 and 2.

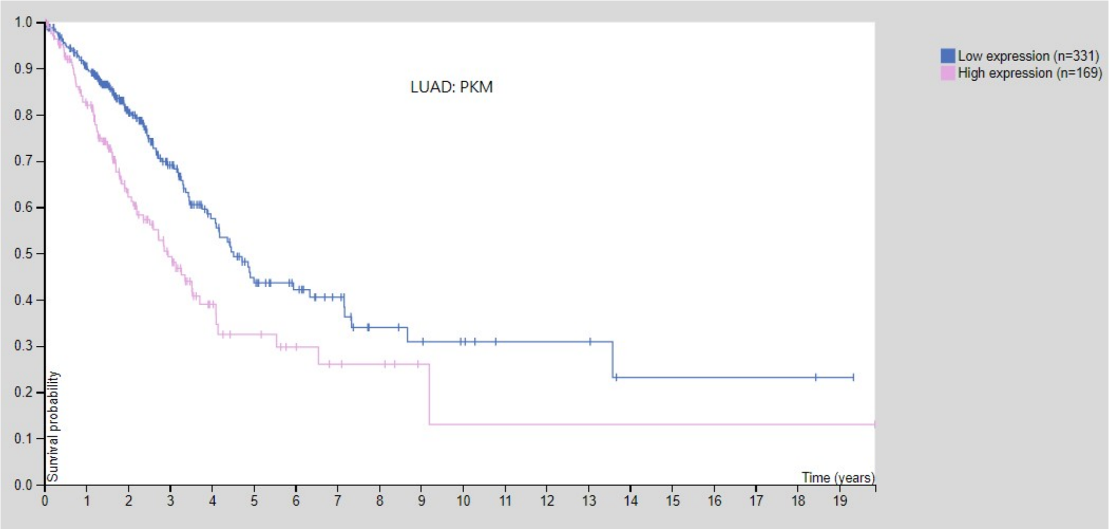

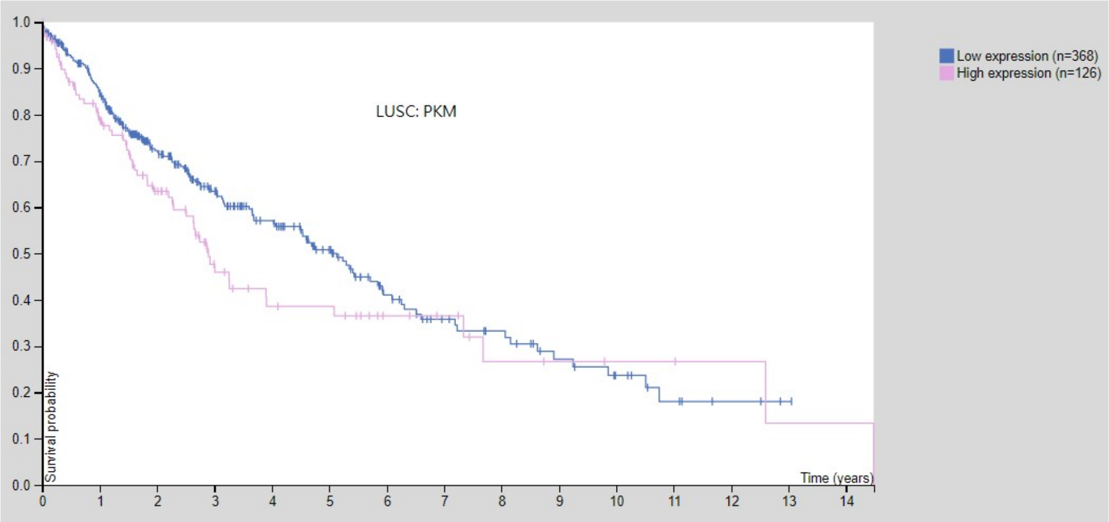

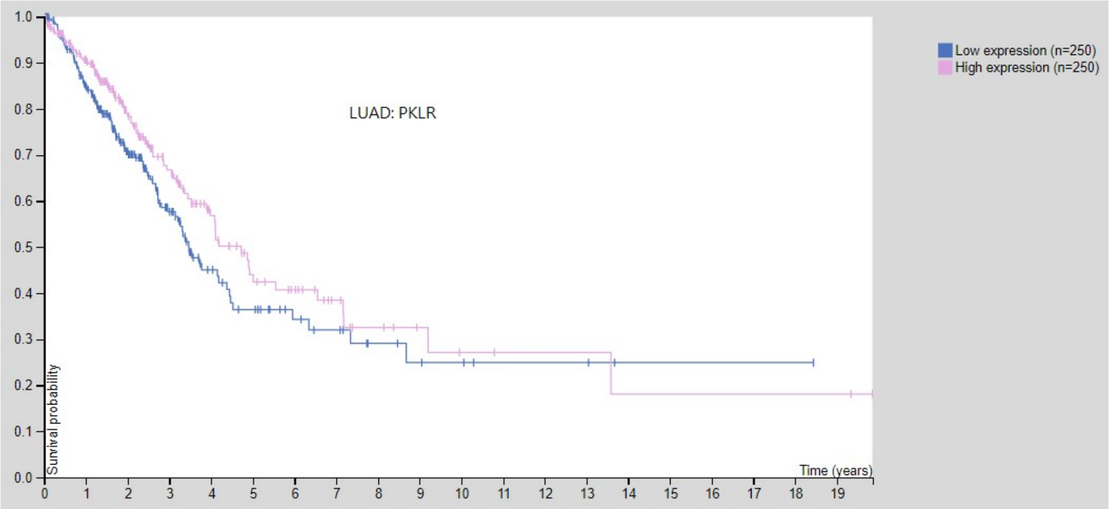
LUAD: PKLR
Low expression (n=250)
High expression (n=250)
Survival probability
Time (years)

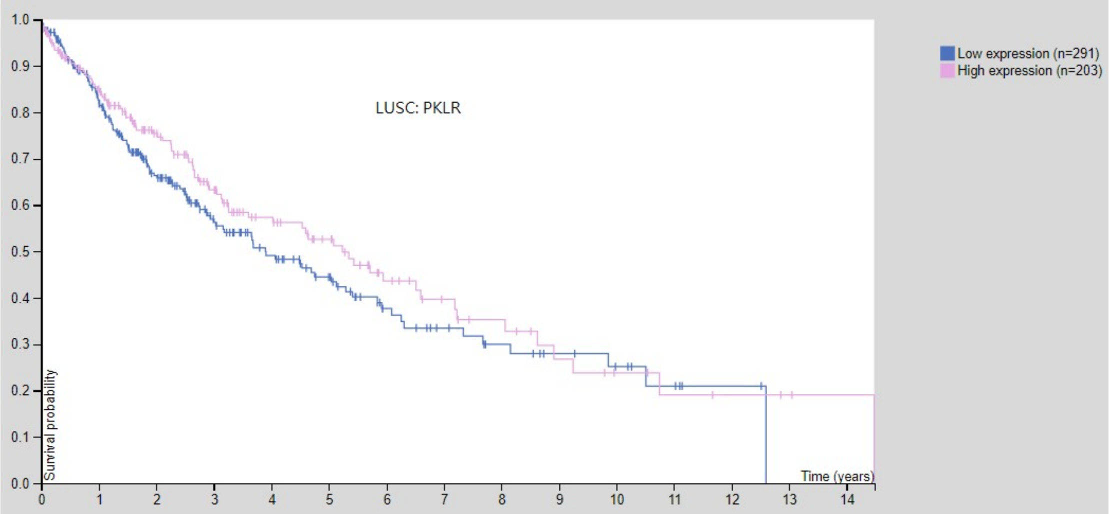
LUSC: PKLR
Low expression (n=291)
High expression (n=203)
Survival probability
Time (years)

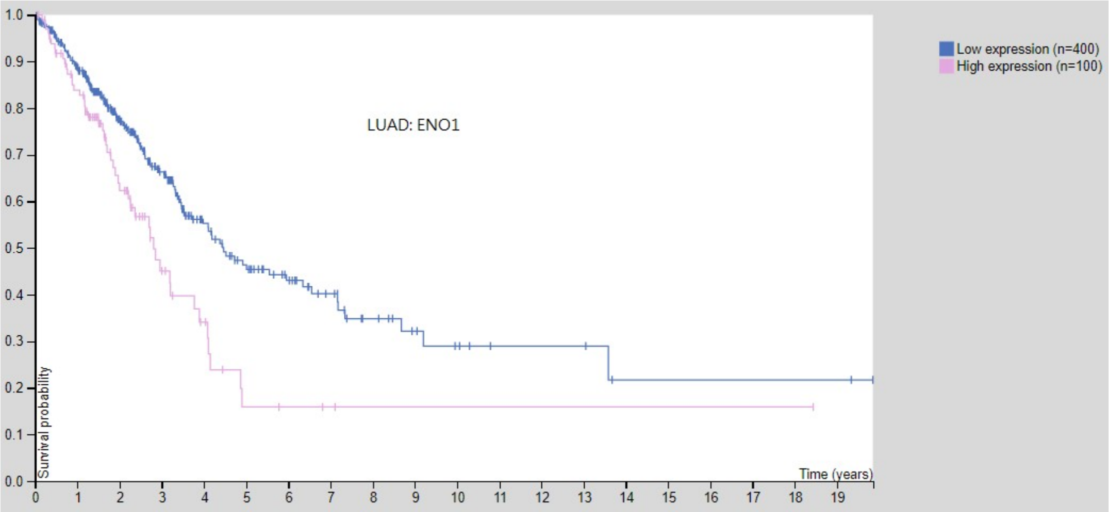

LUAD: ENO1
Low expression (n=400)
High expression (n=100)
Survival probability
Time (years)

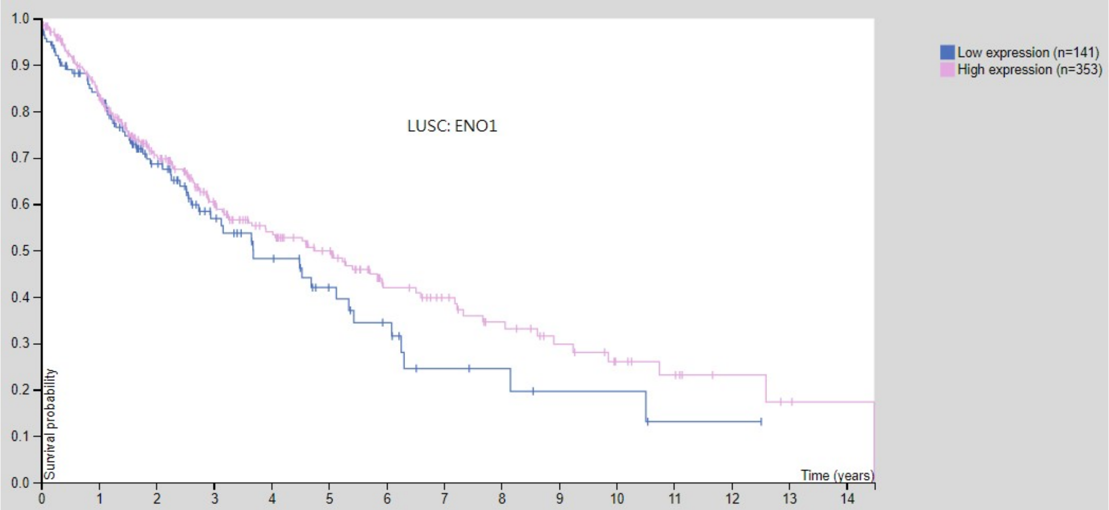

LUSC: ENO1
Low expression (n=141)
High expression (n=353)
Survival probability
Time (years)

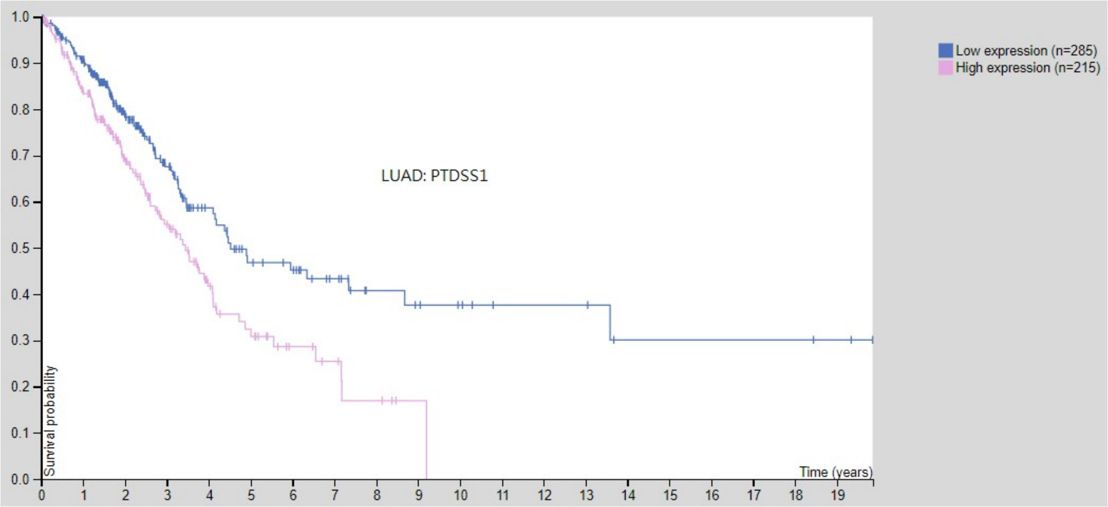

LUAD: PTDSS1
Low expression (n=285)
High expression (n=215)
Survival probability
Time (years)

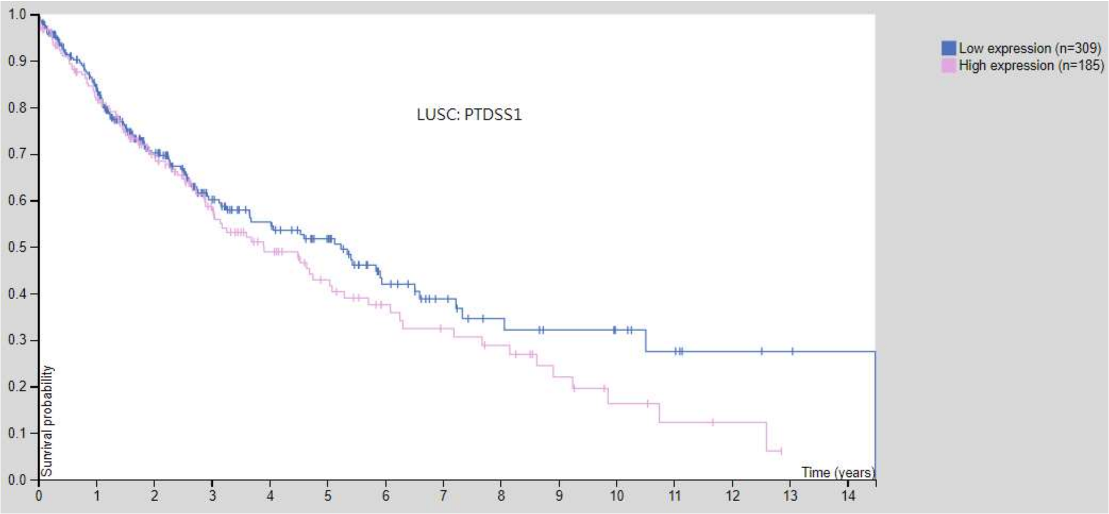

LUSC: PTDSS1
Low expression (n=309)
High expression (n=185)
Survival probability
Time (years)

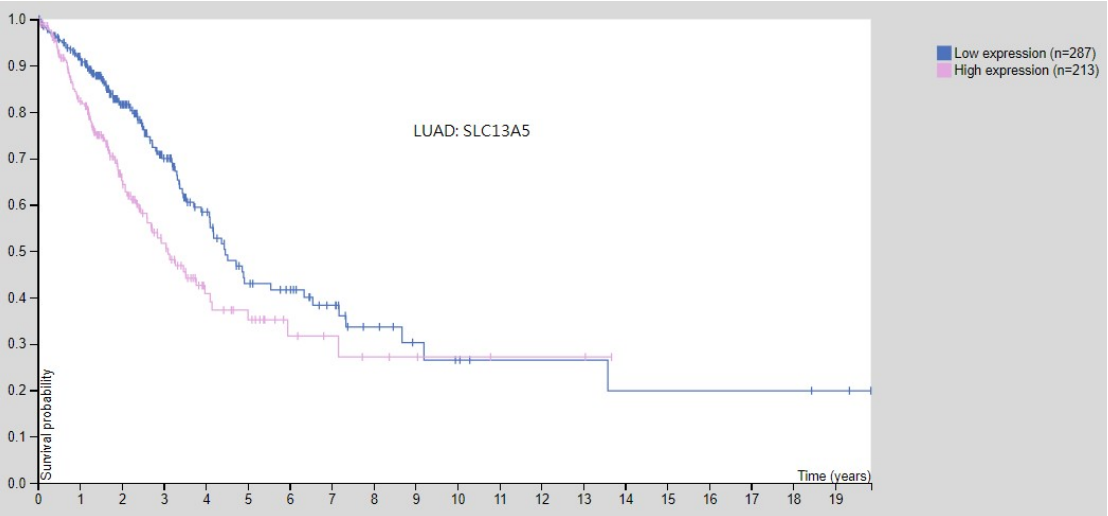
LUAD: SLC13A5
Low expression (n=287)
High expression (n=213)
Survival probability
Time (years)
1.0
0.9
0.8
0.7
0.6
0.5
0.4
0.3
0.2
0.1
0.0
0 1 2 3 4 5 6 7 8 9 10 11 12 13 14 15 16 17 18 19

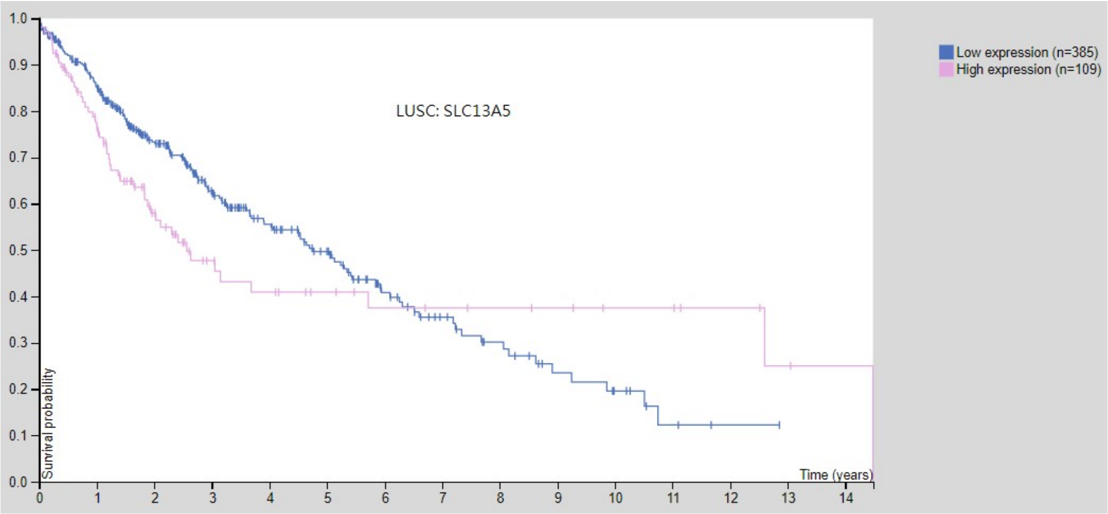
LUSC: SLC13A5
Low expression (n=385)
High expression (n=109)
Survival probability
Time (years)
1.0
0.9
0.8
0.7
0.6
0.5
0.4
0.3
0.2
0.1
0.0
0 1 2 3 4 5 6 7 8 9 10 11 12 13 14

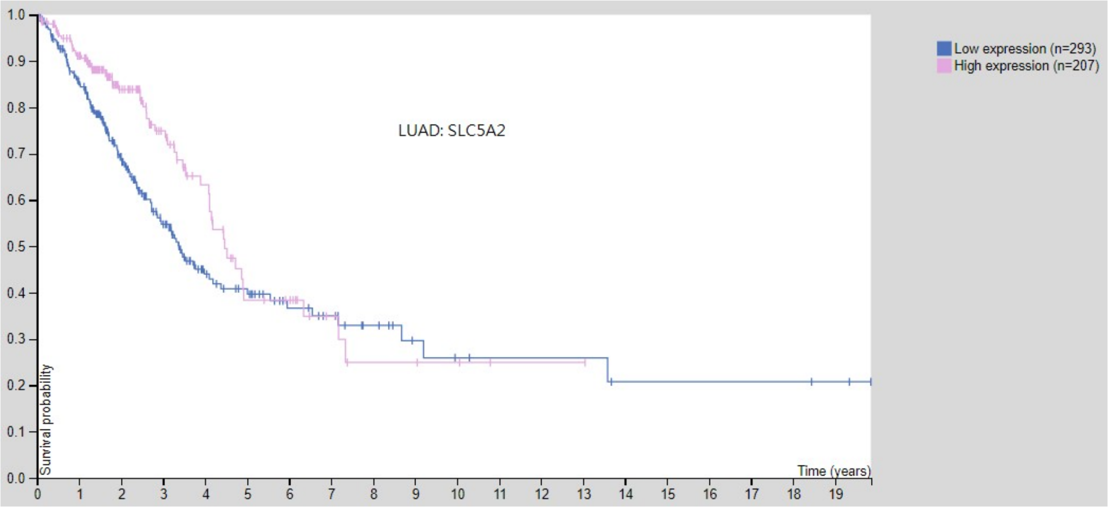
LUAD: SLC5A2
Low expression (n=293)
High expression (n=207)
Survival probability
Time (years)
1.0
0.9
0.8
0.7
0.6
0.5
0.4
0.3
0.2
0.1
0.0
0 1 2 3 4 5 6 7 8 9 10 11 12 13 14 15 16 17 18 19

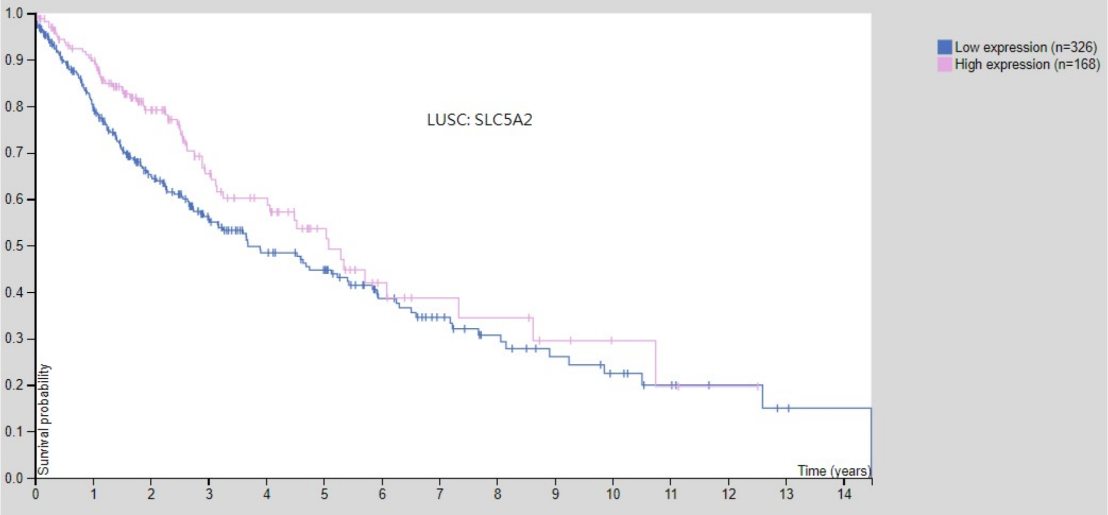
LUSC: SLC5A2
Low expression (n=326)
High expression (n=168)
Survival probability
Time (years)
1.0
0.9
0.8
0.7
0.6
0.5
0.4
0.3
0.2
0.1
0.0
0 1 2 3 4 5 6 7 8 9 10 11 12 13 14

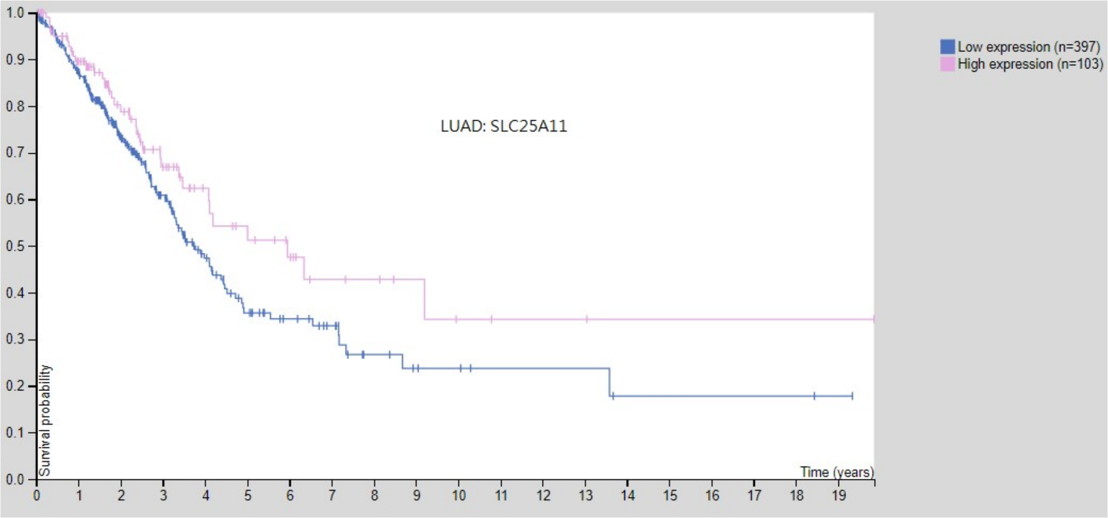

LUAD: SLC25A11
Low expression (n=397)
High expression (n=103)
Survival probability
Time (years)
1.0
0.9
0.8
0.7
0.6
0.5
0.4
0.3
0.2
0.1
0.0
0 1 2 3 4 5 6 7 8 9 10 11 12 13 14 15 16 17 18 19

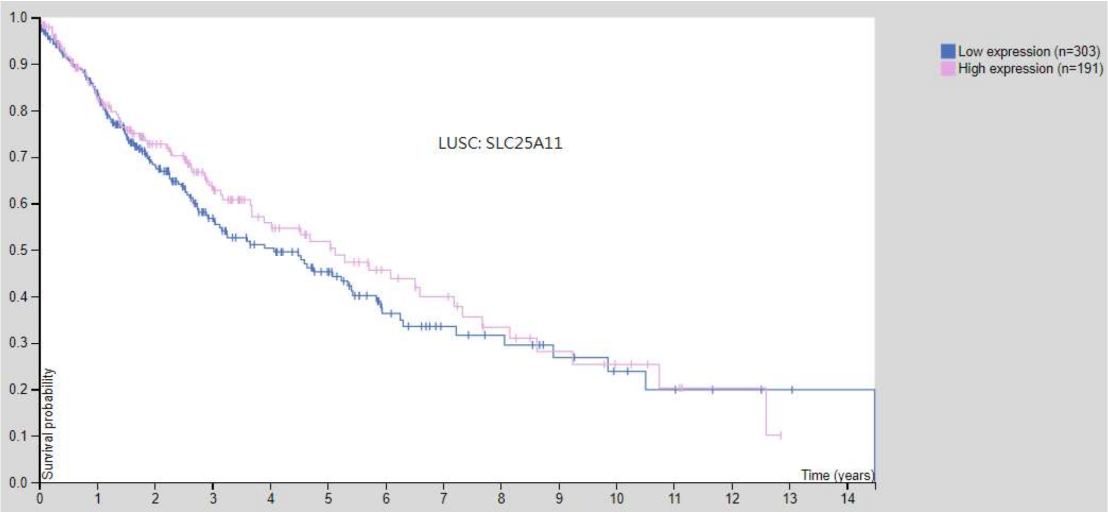

LUSC: SLC25A11
Low expression (n=303)
High expression (n=191)
Survival probability
Time (years)
1.0
0.9
0.8
0.7
0.6
0.5
0.4
0.3
0.2
0.1
0.0
0 1 2 3 4 5 6 7 8 9 10 11 12 13 14

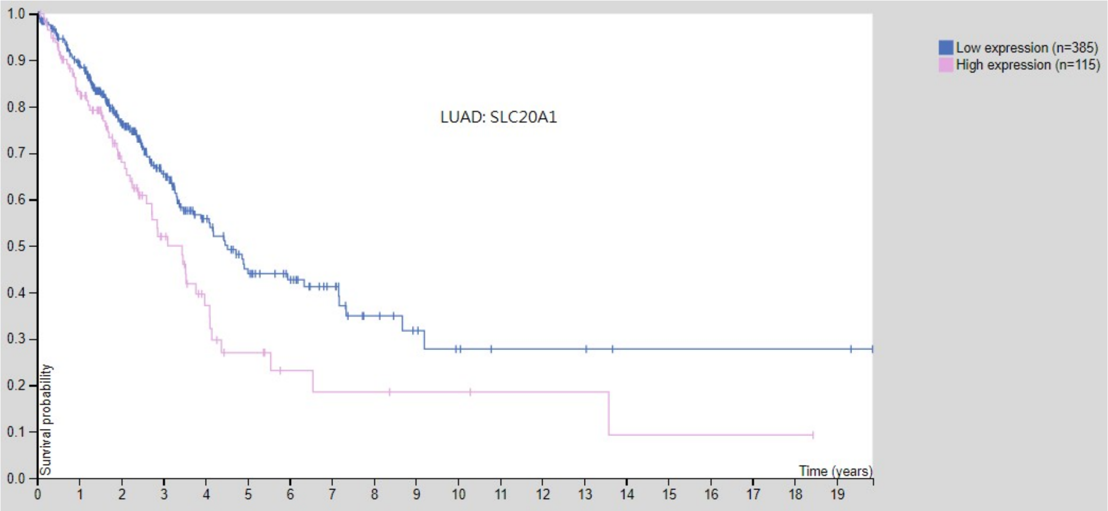
LUAD: SLC20A1
Low expression (n=385)
High expression (n=115)
Survival probability
Time (years)
1.0
0.9
0.8
0.7
0.6
0.5
0.4
0.3
0.2
0.1
0.0
0 1 2 3 4 5 6 7 8 9 10 11 12 13 14 15 16 17 18 19

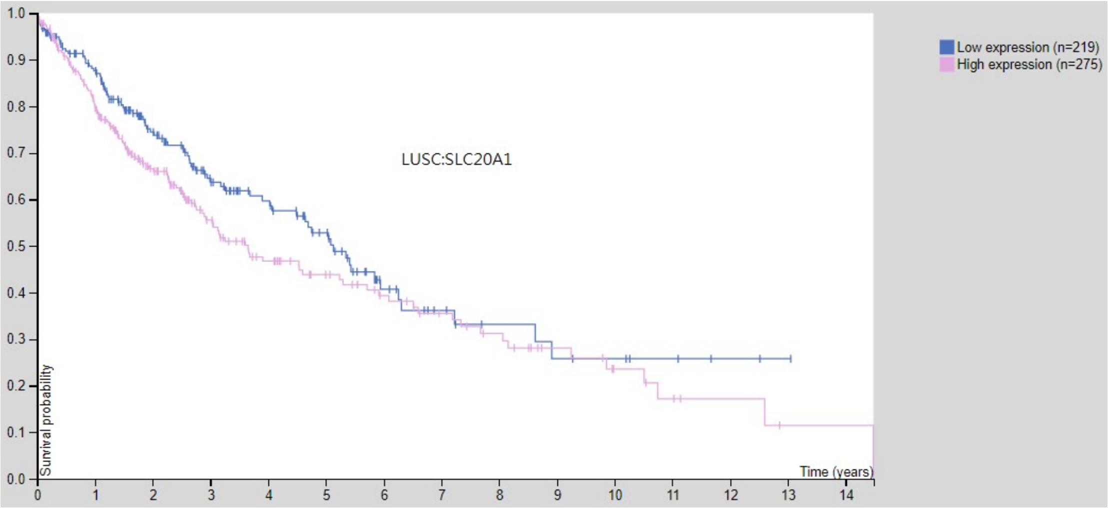
LUSC:SLC20A1
Low expression (n=219)
High expression (n=275)
Survival probability
Time (years)
1.0
0.9
0.8
0.7
0.6
0.5
0.4
0.3
0.2
0.1
0.0
0 1 2 3 4 5 6 7 8 9 10 11 12 13 14

Pathology of SLC22A7 for LUAD is not available.

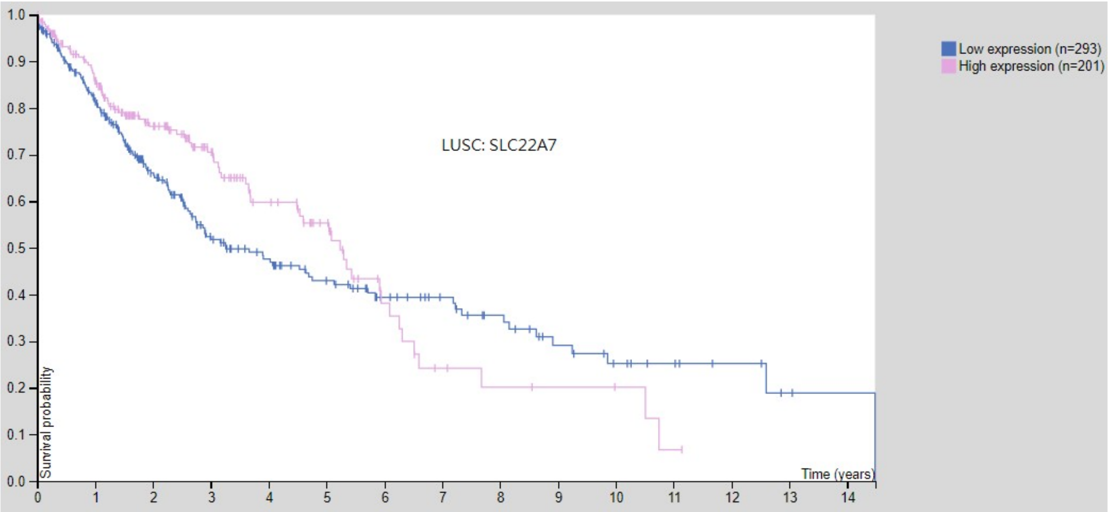

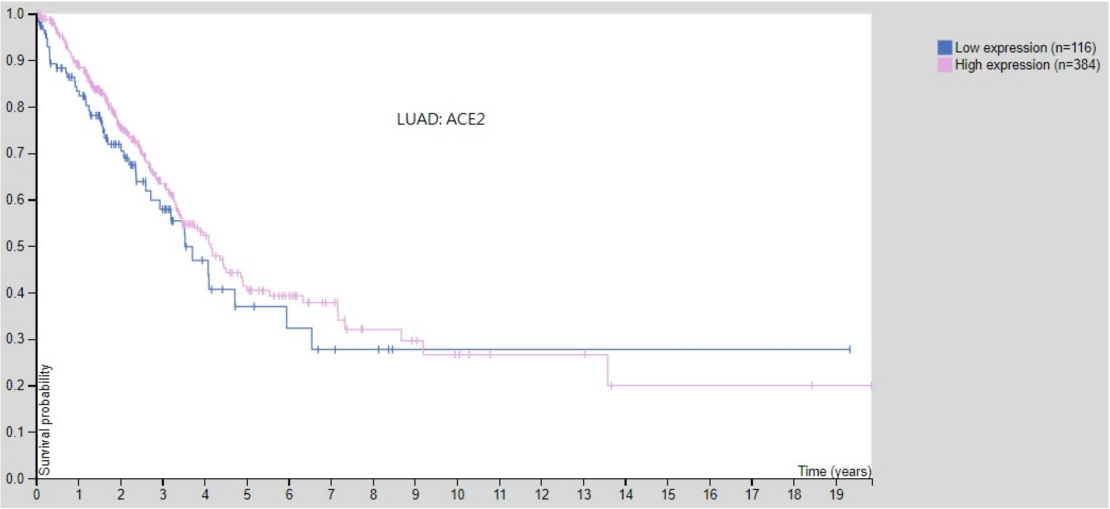

LUAD: ACE2
Low expression (n=116)
High expression (n=384)
Survival probability
Time (years)

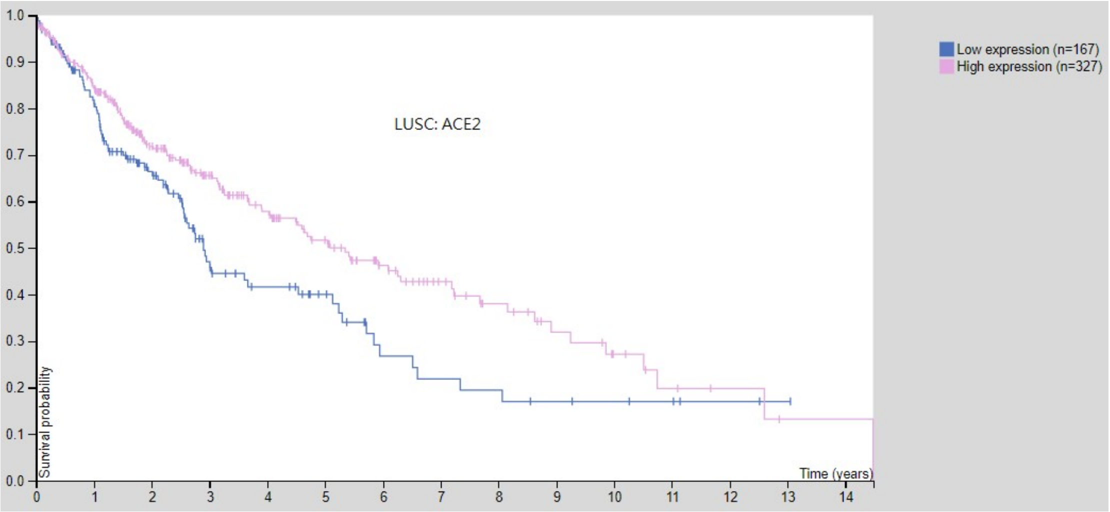

LUSC: ACE2
Low expression (n=167)
High expression (n=327)
Survival probability
Time (years)
