# Supplementary material for: Optimization of a modeling platform to predict oncogenes from genome‐scale metabolic networks of non‐small‐cell lung cancers
Source: FEBS Open Bio. 2021 Jul 20;11(8):2078–94. doi: 10.1002/2211-5463.13231 (PMC8329960; doi:10.1002/2211-5463.13231)
Supplement: Supplementary file 7 — Doc. S7. Similarity ratios and membership grades of the dysregulated genes from Tables 1 and 2 for the LUAD and LUSC GSMNs reconstructed by integrating the iMAT algorithm with data from different databases (TCGA or HPA). [file FEB4-11-2078-s008.pdf]

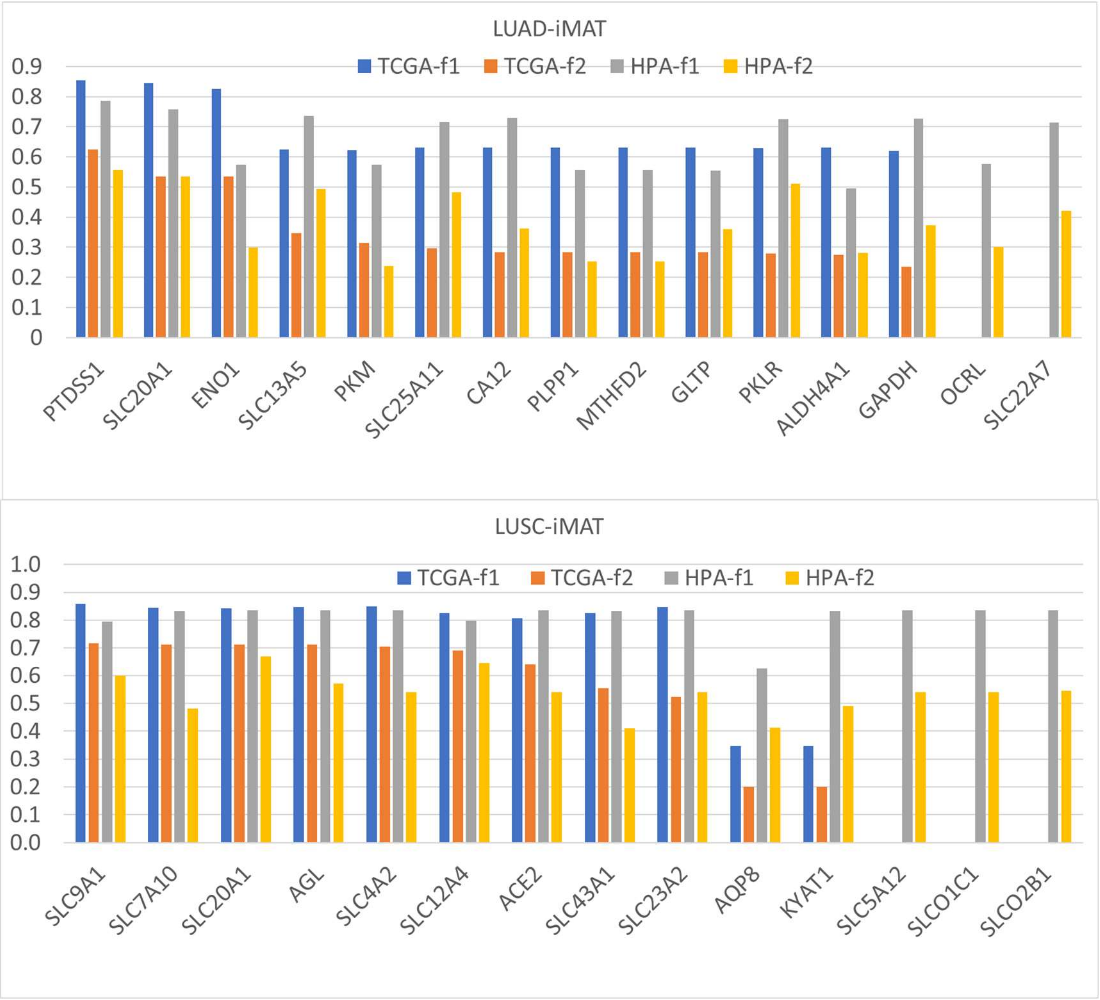

Similarity ratios and membership grades for the dysregulated genes obtained from Table 1 and 2. TCGA/HPA-f1 and TCGA/HPA-f2 denoted as similarity ratio and membership grade are computed using the GSMNs reconstructed by TCGA or HPA database, respectively. The iMAT algorithm was applied to reconstruct GSMN of LUAD and LUSC by integrated TCGA or HPA database, respectively.

Zur, H.; Ruppin, E.; Shlomi, T.; iMAT: an integrative metabolic analysis tool. Bioinformatics, 2010, 26, 24:3140-3142.
